# Tipping the immunostimulatory and inhibitory DAMP balance to harness immunogenic cell death

**DOI:** 10.1038/s41467-020-19970-9

**Published:** 2020-12-07

**Authors:** K. Hayashi, F. Nikolos, Y. C. Lee, A. Jain, E. Tsouko, H. Gao, A. Kasabyan, H. E. Leung, A. Osipov, S. Y. Jung, A. V. Kurtova, K. S. Chan

**Affiliations:** 1 Department of Pathology and Laboratory Medicine, Cedars-Sinai Medical Center, Los Angeles, CA 90048 USA; 2 Graduate Program in Translational Biology and Molecular Medicine, Baylor College of Medicine, Houston, TX 77030 USA; 3 Samuel Oschin Cancer Center, Cedars-Sinai Medical Center, Los Angeles, CA 90048 USA; 4 Graduate Institute of Medical Sciences, Taipei Medical University, Taipei City, Taiwan; 5 Alkek Center for Molecular Discovery, Proteomics Core, Baylor College of Medicine, Houston, TX 77030 USA; 6 Department of Orthopedic Surgery, Baylor College of Medicine, Houston, TX 77030 USA

**Keywords:** Chemotherapy, Pancreatic cancer, Tumour immunology, Bladder cancer, Cell death and immune response

## Abstract

Induction of tumor cell death is the therapeutic goal for most anticancer drugs. Yet, a mode of drug-induced cell death, known as immunogenic cell death (ICD), can propagate antitumoral immunity to augment therapeutic efficacy. Currently, the molecular hallmark of ICD features the release of damage-associated molecular patterns (DAMPs) by dying cancer cells. Here, we show that gemcitabine, a standard chemotherapy for various solid tumors, triggers hallmark immunostimualtory DAMP release (e.g., calreticulin, HSP70, and HMGB1); however, is unable to induce ICD. Mechanistic studies reveal gemcitabine concurrently triggers prostaglandin E_2_ release as an inhibitory DAMP to counterpoise the adjuvanticity of immunostimulatory DAMPs. Pharmacological blockade of prostaglandin E_2_ biosythesis favors CD103^+^ dendritic cell activation that primes a Tc1-polarized CD8^+^ T cell response to bolster tumor rejection. Herein, we postulate that an intricate balance between immunostimulatory and inhibitory DAMPs could determine the outcome of drug-induced ICD and pose COX-2/prostaglandin E_2_ blockade as a strategy to harness ICD.

## Abstract (web summary)

Most chemotherapeutic agents, including gemcitabine, do not elicit immunogenic cell death, a phenomenon associated with the release of damage-associated molecule patterns (DAMPs). Here, the authors show that gemcitabine-treated dying cancer cells express hallmark DAMPs but their immunogenic properties are hindered by the concomitant release of the inhibitory DAMP PGE_2_.

## Introduction

Most anticancer therapies designate tumor cell death as the ultimate biological and therapeutic end goal. Yet, studies are emerging to illuminate the often-overlooked physiological processes resulting from cell death as important factors that influence therapeutic efficacy. During development and homeostasis, billions of cells undergo a highly orchestrated mode of immunologically silent (or tolerogenic) cell death—these dead/dying cells are cleared by innate immune cells without provoking an inflammatory response. In contrast, cells that undergo immunogenic cell death (ICD) during pathogenic or xenobiotic insult, for example, actively potentiate an adaptive immune response to properly resolve these pathologic conditions^1–6^. In the context of anticancer therapeutics, drugs capable of provoking ICD are deemed clinically relevant based on their inherent propensity to augment therapeutic efficacy via recruiting antitumoral immunity^1–6^.

The current established paradigm and defining molecular hallmark of ICD is the release of host-derived, immune-activating molecules known as damage-associated molecular patterns (DAMPs) from dying cells^1–7^. Canonical DAMPs include, but are not limited to: calreticulin (CRT)^8^; heat-shock protein 70 kDa (HSP70)^9^; and high-mobility group box 1 (HMGB1)^10^. During the initial stages of ICD induction, CRT translocates from the endoplasmic reticulum (ER) to the cell surface and functions as an “eat-me” signal^11^. The translocation of HSP70 from the ER to cell-surface during mid-apoptosis^9^ and the extracellular release of HMGB1 during late-apoptosis^10^ serve as host-derived “danger” signals (or immunological adjuvants^3^). These ectopically expressed DAMPs are postulated to function as signal 0 to engage pattern recognition receptors on professional antigen-presenting cells (e.g., dendritic cells^11^) and other innate immune cells^12^. Together with signal 1 (T cell receptor: major histocompatibility complex), signal 2 (co-stimulation), and signal 3 (cytokine), these professional antigen-presenting cells are properly matured and activated—priming an effective CD8^+^ T cell immune response^8–10,13–15^.

Clinically, drug-induced immunogenic cell death is reported to positively correlate with therapeutic response and associate with an enhanced antitumoral CD8^+^ T cell immunity^16–20^. To date, only a handful of anticancer agents are recognized as bona fide ICD-inducers^17,21^; most therapeutics require the assistance of adjuvant drugs to substantiate DAMP release for successful ICD induction^22^. Thus, in contrast to bona fide ICD-inducers, such as anthracyclines (e.g., mitoxantrone)^23–27^, most standard-of-care chemotherapies, including cisplatin^17^ and gemcitabine^28^, are considered non-ICD-inducing as monotherapies. The immunological adjuvanticity of hallmark DAMPs in propagating antitumoral immune response(s) have been extensively studied^1–11^. However, whether chemotherapy-induced DAMP release alone dictates the immunogenic versus tolerogenic fate of cell death is not fully understood.

Here, we pose the induction of prostaglandin E_2_ as an inter-regulatory counterpoise to immunostimulatory DAMPs in the context of chemotherapy-induced ICD. Albeit the success of gemcitabine in inducing hallmark DAMP release, PGE_2_ dominantly skews cell death towards a tolerogenic, rather than immunogenic phenotype. Building on the prior works of others, we define PGE_2_ as an inhibitory DAMP (iDAMP) that converges with immunostimulatory DAMPs as signal 0 to dictate the maturation of dendritic cells and influence CD8^+^ T cell effector function.

## Results

### Gemcitabine potentiates the release of hallmark DAMPs from dying cancer cells

**Fig. 1:**
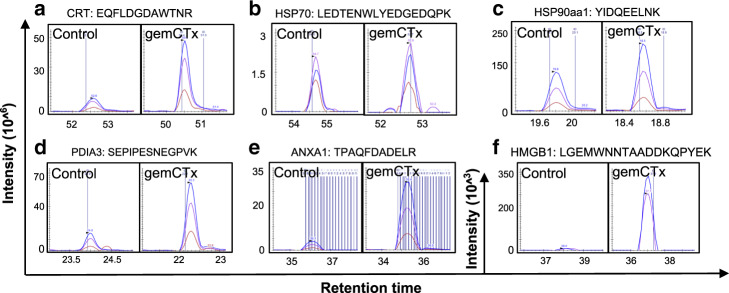
The release of hallmark DAMPs from gemcitabine-induced dying cancer cells. Representative peptide chromatogram illustrating differential enrichment of **a** CRT, **b** HSP70, **c** HSP90aa1, and **d** PDIA3 on the cell surface, as well as **e** ANXA and **f** HMGB1 released into the cultured media by cancer cells (murine G69 bladder cancer cells) treated with gemcitabine (*n* = 2 independent mass spectrometry proteomic profiling experiments). ANXA1 annexin A1, CRT calreticulin, gemCTx gemcitabine chemotherapy-treated, HMGB1 high-mobility group protein box 1, HSP heat-shock protein, PDIA3 protein disulfide isomerase A3. Extracted chromatograms for a representative peptide of each protein was plotted using the Skyline software; precursor (blue color), precursor [M + 1] (purple color), precursor [M + 2] (red color) represent the three isotopic peaks for each indicated peptide.

Most chemotherapeutic agents are non-immunogenic, with only a handful reported to be ICD-inducing^4^. These immunogenic chemotherapies are characterized by their capacity to induce dying cells that (i) are coupled with hallmark DAMP release^3^ and (ii) serve as functional in vivo vaccines to prime an antitumoral CD8^+^ T cell response using a gold-standard vaccination assay^26,27^. Gemcitabine is a commonly prescribed chemotherapy for treating various solid tumors (e.g., bladder and pancreatic cancers). However, whether gemcitabine as a monotherapy potentiates ICD via hallmark DAMP release is limited; a prior study reported that gemcitabine is non-immunogenic^28^. To evaluate this further, we subjected human bladder cancer cells (i.e., T24) to gemcitabine chemotherapy treatment in vitro. Quantitative proteomic profiling of fractionated cellular compartments via mass spectrometry was performed to identify cell surface and extracellularly secreted proteins upon gemcitabine treatment. To categorize relevant proteins, we employed two analytical approaches: (i) proteins sharing similar amino acid sequences, protein domains, or structures and (ii) proteins sharing similar functions. While no structurally similar proteins were revealed by mass spectrometry, we detected cell surface calreticulin (CRT; Fig. 1a, b) and other proteins with known immunological effector function(s)^3^. Intriguingly, a series of proteins classified as hallmark DAMPs were identified in the extensive mass spectrometry protein list. These included cell-surface heat-shock proteins 70 kDa (HSP70) and 90 kDa (HSP90), as well as extracellular high-mobility group protein (HMGP; Fig. 1a). Specific protein enrichment was quantified by grouping unique peptide sequences corresponding to each DAMP and compared to gemcitabine-treated versus untreated cells (Table 1 and Supplementary Fig. 1a). These initial findings challenged the prior study reporting that gemcitabine is unable to promote cell surface DAMP (i.e., CRT) expression to induce ICD in a pancreatic ductal adenocarcinoma (PDAC) model^28^.

**Table 1:** Mass Spectrometry profiling reveals hallmark DAMPs from dying cancer cells after gemcitabine treatment.

Protein name	T24 gemCTx:control (unique peptide no.)	G69 gemCTx:control (iFOT ratio)	Panc02 gemCTx:control (iFOT ratio)	Fraction analyzed
ANXA1	ND	606:285 (2.12)	33.15:ND (33.15)	Cultured media
CRT	10	108:14.8 (7.29)	243:16.4 (14.82)	Cell membrane
HMGP HMGB1	2	13.2:7.4 (1.78)	17.3:ND (17.3)	Cultured media
HSP70	8	5.3:3.1 (1.71)	14.3:4.5 (3.18)	Cell membrane
HSP90	29	142:103 (1.38)	138.5:93.6 (1.48)	Cell membrane
PDIA3	ND	147:28.5 (5.16)	247.5:12.2 (20.29)	Cell membrane

A table summarizing the detection of classical DAMPs from gemcitabine-induced dying cancer cells, via mass spectrometry (MS) proteomic profiling. Quantification of peptide enrichment (gemCTx- vs. non-treated control) by either (i) number of unique peptide sequences (T24 cells) or (ii) average iFOT ratio (G69 and Panc02 cells; *n* = 2 independent experiments) for each of the detected proteins.

*ANXA1* annexin A1, *CRT* calreticulin, *gemCTx* gemcitabine chemotherapy-treated, *HMGB1* high-mobility group protein box 1, *HSP* heat-shock protein, *ND* not detected, *PDIA3* protein disulfide isomerase A3.

Since T24 is a human bladder cancer cell line and does not allow for the functional evaluation of ICD in immunocompetent hosts, we developed a murine bladder cancer model (designated hereinafter as G69). The advent of this tool permits for the functional evaluation of bladder cancer ICD in syngeneic, immunocompetent hosts in vivo (Supplementary Fig. 2). As a complementary approach, we also utilized a murine PDAC model that was previously established for investigating ICD (i.e., Panc02^28^), as proof-of-concept to generalize our findings in a different tumor type. Proteomic profiling was performed using the cell-surface fraction and cultured medium of gemcitabine- or vehicle-treated G69 and Panc02 cells in vitro (Table 1). As demonstrated in Fig. 1a–e, in addition to the enriched expression of cell surface CRT, HSP70, and HSP90, gemcitabine treatment also induced the expression of disulfide isomerase family A member 3 (PDIA3)^29^, as well as the extracellular release of high-mobility group protein B1 (HMGB1) and annexin A1 (ANXA1)^30^ into the cultured media (Fig. 1e–g and Supplementary Fig. 1b). Here, proteomic enrichment of DAMPs was quantified using the total unique protein counts normalized to total fraction counts and unique peptide sequences (iFOT; Fig. 1a; additional representative peptide peaks are presented as raw data in Supplementary Fig. 1). Collectively, these profiling results from three independent cancer models convincingly demonstrated that gemcitabine, as a monotherapy, potentiates hallmark DAMP release as a generalized phenomenon—the current molecular prerequisite of ICD.

### Hallmark DAMP release is insufficient to induce immunogenic cell death

**Fig. 2:**
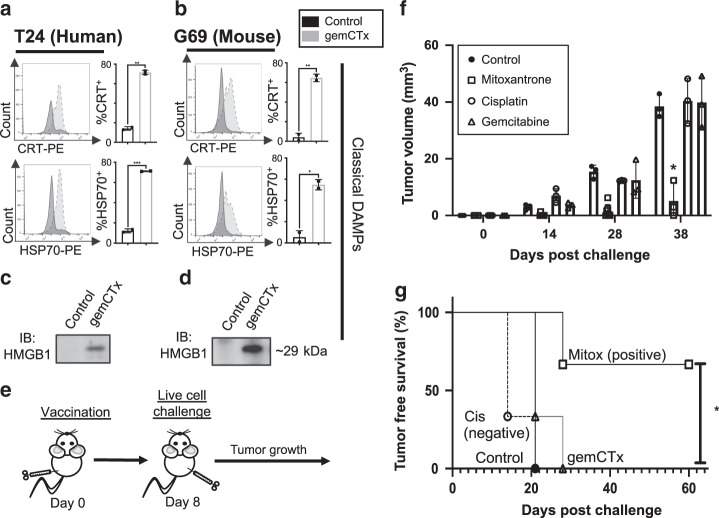
Hallmark DAMP release is insufficient to induce immunogenic cell death. **a**, **b** Flow cytometry analysis and validation of DAMPs (i.e., CRT and HSP70) on the cell surface of human T24 and murine G69 bladder cancer cells treated with gemcitabine in vitro (representative plot shown with two technical replicates of *n* = 3 independent experiments; example gating depicted in Supplementary Fig. 3). **c**, **d** Western blot of HMGB1, an extracellular DAMP, released into cultured media that were collected from gemcitabine-treated human T24 cells and murine G69 cells, respectively. **e** A schematic depiction of the in vivo vaccination assay treatment schedule for assaying immunogenic cell death. **f** Tumor volume and **g** corresponding tumor-free survival Kaplan–Meier plot (*n* = 3 mice per vaccination group; tumor volume calculation as indicated in Methods section) resulting from mice vaccinated with dying G69 cells via various chemotherapy and subsequently challenged with live G69 murine bladder cancer cells. cis cisplatin, gem gemcitabine, mitox mitoxantrone. Statistics: two-tailed, unpaired T test (**a**; *p* = 0.0013 [top] and *p* = 0.0006 [bottom]) and (**b**; *p* = 0.0044 [top] and *p* = 0.0123 [bottom]); two-tailed, two-way ANOVA-Tukey’s multiple comparisons test (**f**; *p* < 0.0240); Kaplan–Meier survival analysis using Mantel–Cox test (**g**; *p* = 0.0123); and where appropriate, data are presented as mean values ±SEM.

**Fig. 3:**
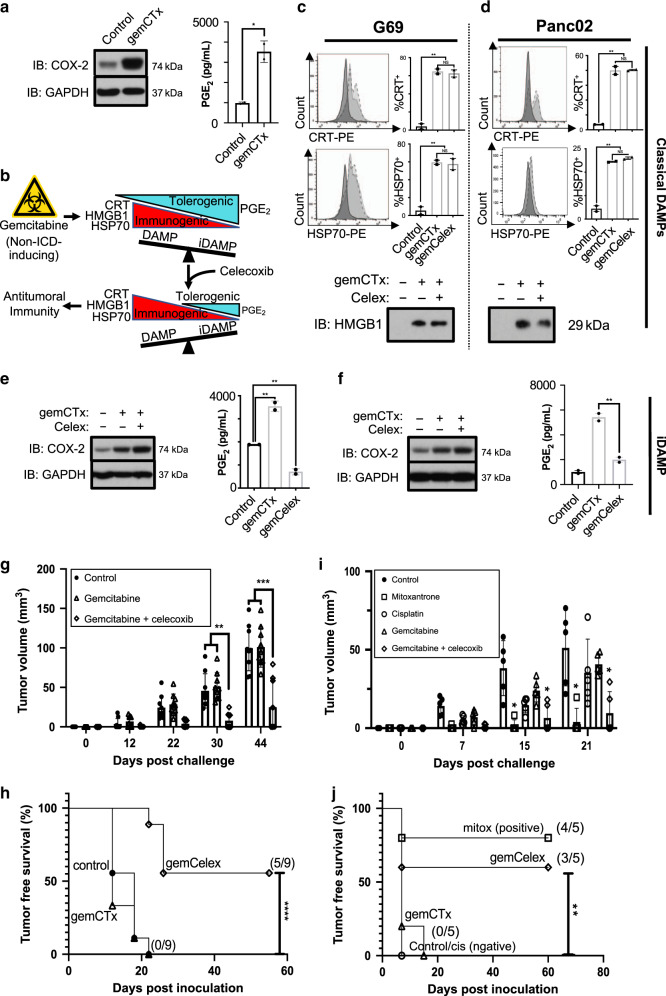
Prostaglandin E_2_ release hinders ICD-induction by gemcitabine. **a** Western blot analysis of COX-2 and corresponding ELISA of PGE_2_ from G69 cells treated with gemcitabine in vitro (representative plot shown with two technical replicates of *n* = 3 independent experiments). **b** A schematic depicting the proposed interventional strategy that exploits the immunostimulatory/inhibitory DAMP balance to harness immunogenic cell death. **c**, **d** Flow cytometry of cell surface CRT and HSP70 of single cells (example gating depicted in Supplementary Fig. 3), as well as western blot of HMGB1 from G69 and Panc02 cells treated with gemcitabine plus celecoxib to achieve iDAMP blockade (representative plot shown with two technical replicates of *n* = 3 independent experiments). **e**, **f** Western blot of COX-2 and ELISA of PGE_2_ from murine G69 and Panc02 cells to demonstrate the efficacy of celecoxib in abrogating gemcitabine-induced PGE_2_/ iDAMP release in vitro (representative plot shown with two technical replicates of *n* = 3 independent experiments). **g** Temporal changes in tumor volume and **h** tumor-free survival of mice after challenge (*n* = 9 per treatment group) resulting from a gold-standard vaccination assay. **i** Temporal changes in tumor volume and **j** tumor-free survival of mice after challenge (*n* = 5 per treatment group) resulting from a gold-standard vaccination assay using the murine Panc02 PDAC model. celex celecoxib, CTx chemotherapy, gem gemcitabine. Statistics: two-tailed, unpaired T test (**a**; *p* = 0.0107); two-tailed, one-way ANOVA-Tukey’s multiple comparisons test (**c**; *p* = 0.002 [top] and *p* = 0.008 [bottom]), (**d**; *p* = 0.0011 [top] and *p* = 0.0007 [bottom]), (**e**; **p = 0.0076 and **p = 0.0028), (**f**; *p* = 0.0033), (**g**; **p = 0.015 and ***p = 0.0003), and (**i**; *p < 0.0486); Kaplan–Meier survival analysis using Mantel–Cox test (**h**; *p* = 0.0001) and (**j**; *p* = 0.004); and where appropriate, data are presented as mean values ±SEM.

To validate the mass spectrometry results in Fig. 1, we profiled for cell surface and extracellular DAMPs using flow cytometry and western blot, respectively. Reflective of the proteomics analyses, both human T24 and murine G69 bladder cancer cells displayed enrichment of cell surface CRT and HSP70 after 48 h of gemcitabine treatment in vitro, when compared to vehicle-treated control cells (Fig. 2a). Importantly, the cell surface expression of CRT was present in DAPI-negative, membrane impermeable (i.e., live) cells (Supplementary Fig. 3a–c). While the bona fide ICD-inducing chemotherapy, mitoxantrone (anthracycline^24^), potentiated substantial cell surface CRT expression in all three models, cisplatin (a non-ICD-inducing chemotherapy), in contrast, failed to promote significant cell surface CRT expression^8,24^ (Supplementary Fig. 3a–c). These results further corroborate the previous works of others, demonstrating the inadequacy of cisplatin to promote cell surface CRT translocation^8,23^. As for extracellular DAMPs, western blot analyses confirmed the release of HMGB1 into the culture media by both G69 and Panc02 cancer cells treated with gemcitabine for 48 h (Fig. 2b and Supplementery Fig. 3d, e). Additionally, gemcitabine treatment also prompted the release of a non-protein extracellular DAMP, ATP^25^ (Supplementary Fig. 3f, g).

The release of both “eat-me” and “danger” DAMP signals from multiple cancer models led us to re-evaluate the immunogenic properties of gemcitabine using the gold-standard in vivo vaccination assay^26,27^. First, murine G69 cancer cells were treated in vitro with IC-50 dose of chemotherapy for 24 h to initiate cell death. Next, either dying cells exposed to mitoxantrone (positive control)^20^, cisplatin (negative control)^8,23^, or gemcitabine (unknown), were injected subcutaneously into age-matched syngeneic hosts as vaccines in the left hind flank (*n* = 3 mice per vaccine group). PBS was used as the non-treated control vaccine. Seven days post-vaccination, mice were challenged with live G69 cells via subcutaneous inoculation on the opposing, right lower flank (Fig. 2e). Vaccination with cancer cells exposed to ICD-inducers (e.g., mitoxantrone^20^) are expected to successfully prevent tumor engraftment when challenged, by priming an antigen-specific CD8^+^ T cell response (Supplementary Fig. 4a). Conversely, mice vaccinated with cells exposed to non-ICD-inducers (e.g., cisplatin^8,23^) are expected to fail in tumor rejection (Supplementary Fig. 4b). While the bona fide ICD-vaccine (i.e., mitoxantrone-treated G69 cells) successfully promoted tumor rejection in mice a week after, the vaccine utilizing cisplatin (negative control) failed (Fig. 2f, g). These results corroborate the previous works of others: in our bladder cancer model, mitoxantrone is a conserved ICD-inducer, while cisplatin remains a non-ICD-inducer. Altogether, these results validated G69 as a viable study model for investigating ICD in bladder cancer.

Despite the induction of hallmark DAMPs in vitro, mice vaccinated with gemcitabine-treated cancer cells failed to present signs of successful immunization upon challenge. All tumors engrafted became palpable within 28 days post challenge (Fig. 2f, g). Moreover, there were no statistical difference in tumor-free survival (Fig. 2f), nor tumor volume (Fig. 2g) between the negative controls (i.e., cisplatin and PBS) and gemcitabine vaccine groups. Therefore, the induction of hallmark DAMPs by gemcitabine is not sufficient to induce ICD in a biological setting.

### Prostaglandin E_2_ release hinders ICD-induction by gemcitabine

The inability of the gemcitabine-treated G69 bladder cancer cells to potentiate proper immunization—despite hallmark DAMP release—led us to hypothesize that immunostimulatory DAMPs are unlikely the only molecular determinant governing immunogenic cell death^3–5,31,32^. A recent study has implicated prostaglandin E_2_ (PGE_2_) as an inhibitory DAMP (iDAMP) in the context of mechanical stress (i.e., repeated freeze-and-thaw) using macrophages^33^. Independently, we have previously reported that gemcitabine-cisplatin chemotherapy induces cyclooxygenase-2 (COX-2) expression, resulting in the release of PGE_2_ from dying bladder tumor cells^34^. Therefore, we sought to investigate whether PGE_2_ could function as an iDAMP in this physiological response to chemotherapy treatment.

First, we investigated whether gemcitabine treatment alone potentiates the biosynthesis and release of PGE_2_ via upregulating COX-2 expression^34^. As shown, gemcitabine treatment of human T24 (Supplementary Fig. 5a) and murine G69 (Fig. 3a and Supplementary Fig. 5b) bladder cancer cells in vitro resulted in increased COX-2 expression, as well as significant PGE_2_ release into cultured media. Since both immunostimulatory DAMPs and iDAMP were released as a result of gemcitabine treatment, we hypothesized that the concurrent release of PGE_2_ counteracts the adjuvanticity of immunostimualtory DAMPs, and thus, prevent functional ICD.

To experimentally test this hypothesis, we utilized celecoxib drug treatment—a selective COX-2 molecular inhibitor—as a pharmacological approach to preclude PGE_2_ biosynthesis. We rationalized that the addition of celecoxib to gemcitabine treatment will alleviate the immunosuppressive constraints imposed by PGE_2_, and thereby, tip the balance in favor of immunostimulatory DAMPs to propagate an antitumoral response (Fig. 3b). To validate pharmacological COX-2/PGE_2_ blockade specifically targets the iDAMP without affecting immunostimulatory DAMPs, we treated murine G69 and Panc02 cancer cells with gemcitabine ± celecoxib in vitro. Low dose celecoxib treatment did not significantly impact the rate of cell death (Supplemental Fig. 6a, b), expression of cell-surface CRT and HSP70, nor the extracellular release of HMGB1 into cultured media by both G69 (Fig. 3c and Supplementary Fig. 6c) and Panc02 (Fig. 3d and Supplementary Fig. 6c) cells. As expected, celecoxib significantly diminished the release of PGE_2_ into cultured media by both G69 (Fig. 3e and Supplementary Fig. 6d) and Panc02 cells (Fig. 3f and Supplementary Fig. 6d), when compared to gemcitabine-treated cells. These results indicate that pharmacological COX-2/PGE_2_ blockade selectively inhibits iDAMP release without affecting immunostimulatory DAMP expression.

To evaluate the functional significance of iDAMP blockade in the context of ICD, we repeated the vaccination assay using G69 cells with the addition of a gemcitabine+celecoxib (i.e., gemCelex) vaccination arm. Mice were vaccinated with either PBS (non-treated control) or gemcitabine pre-treated cells ± celecoxib drug treatment (test groups; *n* = 9 mice per treatment arm). As expected, mice that were vaccinated with either PBS or gemcitabine-treated G69 cell vaccines developed palpable tumors within 28 days post challenge. Remarkably, mice that received the gemCelex (i.e., COX-2/PGE_2_ blockade) vaccination displayed a significantly heightened antitumoral response, with hindered tumor growth (Fig. 3g; *p* < 0.05 post 28 days) and engraftment (Fig. 3h; *p* < 0.0001 post 50 days). Next, we interrogated whether the effects of COX-2/PGE_2_ blockade (Fig. 3g, h) is recapitulated in Panc02 cells using the same vaccination assay. In Panc02 cells, COX-2/PGE_2_ blockade significantly augmented the capacity of gemcitabine to potentiate ICD, phenocopying that of the G69 bladder cancer model (Fig. 3i, j). Difference in tumor volume post-engraftment became statistically significant by day 15 post-challenge (Fig. 3i; *p* < 0.05; *n* = 5 mice per group). And as expected, vaccination using mitoxantrone resulted in the greatest tumor rejection (4/5), while the control (PBS), cisplatin and gemcitabine groups resulted poorly (0/5; Fig. 3j). Corroborating our findings in G69 (Fig. 3g, h), the gemCelex vaccine group using Panc02 also displayed enhanced tumor rejection (3/5; Fig. 3j). These results support the notion that ICD is governed by an intricate balance between immunostimulatory and inhibitory DAMP, which is generalizable in different tumor types. Current strategies focus on substantiating immunostimulatory DAMP release to potentiate ICD^22^ without considering the existence or manipulation of inhibitoy DAMP release, underscoring the distinctiveness of the current approach.

### An inhibitory signal 0 counteracts immunostimulatory DAMPs to mitigate immunogenic dendritic cell maturation

Dendritic cells (DCs) are quintessential professional antigen-presenting cells that excel at antigenic cross-presentation and cross-priming of CD8^+^ T cells^35,36^. And thus, the vaccination assay is conceptualized to rely on the successful activation/maturation of DCs for mobilizing antitumoral CD8^+^ T cells to reject tumor engraftment^26,27^. Growing evidence suggests that the specialized CD103^+^ classical DC 1 (cDC1) subset is adept at antigenic cross-presentation and CD8^+^ T cell cross-priming^37–40^ Here, we implemented bone marrow-derived CD103^+^ DC (CD103^+^ BMDC) cultures as a pertinent model for mechanistic study. CD103^+^ BMDCs were generated from 8-week-old mice bone marrow as previously described^41^. We successfully differentiated CD11c^+^CD103^+^XCR1^+^MHCII^+^ DCs from ex vivo bone marrow culture supplemented with fms tyrosine related kinase 3 ligand (FLT3L) and granulocyte–macrophage colony-stimulating factor (GM-CSF). These DCs expressed elevated mRNA levels of basic leucine zipper ATF-Like transcription factor 3 (*Batf3*) and interferon regulatory factor 8 (*Irf8*) transcription factors^42,43^, as well as the hallmark antigen-receptor *Clec9a*^44^ (Supplementary Fig. 7), confirming their characteristics as cDC1 subset for further experimentation^45–47^.

**Fig. 4:**
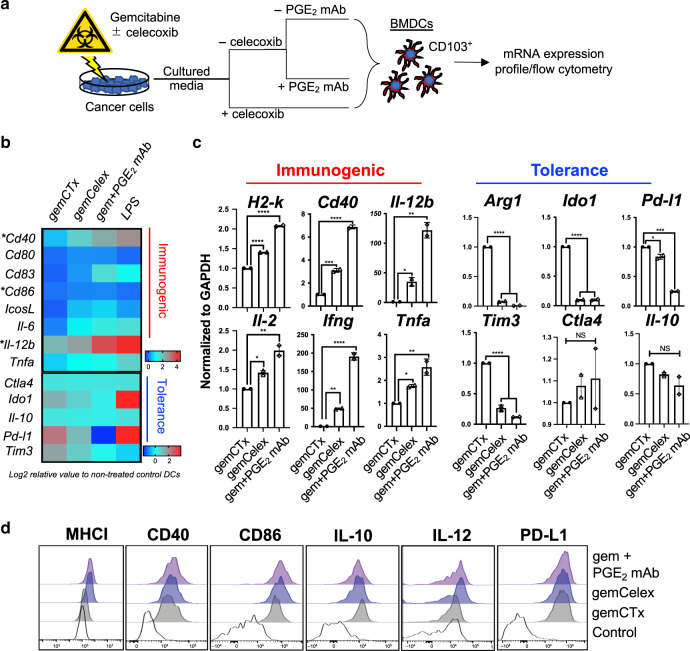
An inhibitory signal 0 counteracts DAMPs to mitigate immunogenic dendritic cell maturation. **a** A schematic depicting the workflow utilized to assay CD103^+^ BMDC activation: CD103^+^ BMDCs were incubated with cultured media from cancer cells pre-treated with gemcitabine ± iDAMP blockade in vitro. PGE_2_ neutralizing antibody or celecoxib were implemented as independent approaches to block PGE_2_ action. **b** Heat-map derived from Fluidigm Biomark™ analyzing surrogate genes representing CD103^+^ BMDC activity that were collected from **a**. Log2 values were calculated from normalized ct values of non-treated CD103^+^ BMDCs as baseline control. **c** qPCR validating genes associated with immunogenic versus tolerogenic dendritic cells. Relative mRNA expression was normalized to *Gapdh* and to gemCTx-treated CD103^+^ BMDCs (representative plot shown with two technical replicates of *n* = 3 independent experiments). **d** Representative flow cytometry histogram plots of CD103^+^ BMDCs 24 h post-cultured media treatment (*n* = 3 independent experiments; example gating depicted in Supplementary Fig. 7). mAb monoclonal antibody, NS statistically non-significant. Statistics: two-tailed, one-way ANOVA-Tukey’s multiple comparisons test (*H2-k*; *p* < 0.0001), (*Cd40*; ***p = 0.0002 and ****p < 0.0001), (*Il-12b*; *p = 0.0475 and **p = 0.0013); (*Il-2*; *p = 0.323 and **p = 0.0069), (*Ifng*; **p = 0.0017 and ****p < 0.0001), (*Tnfa*; *p = 0.0347 and **p = 0.0024), (*Arg1*; *p* < 0.0001), (*Ido1*; *p* < 0.0001), (*Pd-l1*; *p = 0.0103 and ***p = 0.0001), (*Tim3*; *p* < 0.0001); and where appropriate, data are presented as mean values ±SEM.

Next, we assessed the immunomodulatory effects of inhibitory DAMP on CD103^+^ BMDC maturation, by treating CD103^+^ BMDCs with cultured media obtained from gemcitabine (gemCTx)-treated G69 cells ± COX-2/PGE_2_ blockade (Fig. 4a). COX-2/PGE_2_ blockade was achieved through two approaches: (i) concurrent celecoxib drug treatment together with gemcitabine or (ii) monoclonal PGE_2_ neutralizing antibody to sequester PGE_2_ in cultured media. These CD103^+^ BMDC were treated with cultured media for 6 h and harvested for mRNA expression profiling (Fig. 4a). To evaluate the effect(s) of COX-2/PGE2 blockade on skewing CD103^+^ BMDC maturation towards an immunogenic phenotype, a high-throughput qPCR-based platform (Fluidigm Biomark) and conventional qPCR were independently employed to analyze the cultured media-treated CD103^+^ BMDCs.

COX-2/PGE_2_ blockade skewed CD103^+^ BMDCs toward an immunogenic phenotype^48^ (Fig. 4b): confirmed by elevated mRNA expression of (i) *H2-k* (i.e., MHCI); (ii) canonical DC co-stimulatory receptor *Cd40*^49^; and (iii) T-cell polarizing cytokines *Tnfa, Il-12b*, and *Ifng*^50^ (Fig. 4b, c). In contrast, CD103^+^ BMDCs without COX-2/PGE_2_ blockade exhibited a tolerogenic phenotype^48,51^ (Fig. 4b), characterized by the expression of: (i) *Arg1*, a gene important in diminishing T cell activity^52,53^; (ii) *Ido1*, a gene important in preventing T cell expansion/memory formation^54,55^; (iii) *Pdcd1lg*, a gene important form promoting T cell anergy^56,57^; and (iv) *Tim3*, an inhibitory receptor on dendritic cells^58,59^. However, other immunosuppression associated genes—e.g., inhibitory receptor *Ctla4* and inhibitory cytokine *Il-10*—were unaffected (Fig. 4c).

The above differential mRNA expressions (Fig. 4b, c) were further validated at the protein level using flow cytometry. At 6 h post cultured media treatment (where mRNA was analyzed), the protein expression of MHCI, MHCII, and CD40 in CD103^+^ BMDCs were only marginally higher in the COX-2/PGE_2_-blockade groups (Supplementary Fig. 8a). Remarkedly, at 24 h post-treatment, CD103^+^ BMDCs displayed significant elevation of major active marks for DCs in the COX-2/PGE_2_-blockade groups. These included MHCI, MHCII, CD40, CD86, and IL-12 protein expression (Fig. 4d and Supplementary Fig. 8b). The tolerogenic cytokine, IL-10, was significantly reduced as a result of COX-2/PGE_2_ blockade (Fig. 4d). The increased protein expression of MHCI, CD40, and IL-12 (Fig. 4d and Supplementary Fig. 8b) were reflective of the increased mRNA expression observed in Fig. 4b, c. Collectively, these findings support PGE_2_ as a functional iDAMP (or inhibitory signal 0) that counteracts hallmark DAMPs in regulating the immunogenic activation/maturation of CD103^+^ BMDCs.

### COX-2/PGE_2_ blockade promotes priming of a CD8^+^ Tc1-mediated immune response

**Fig. 5:**
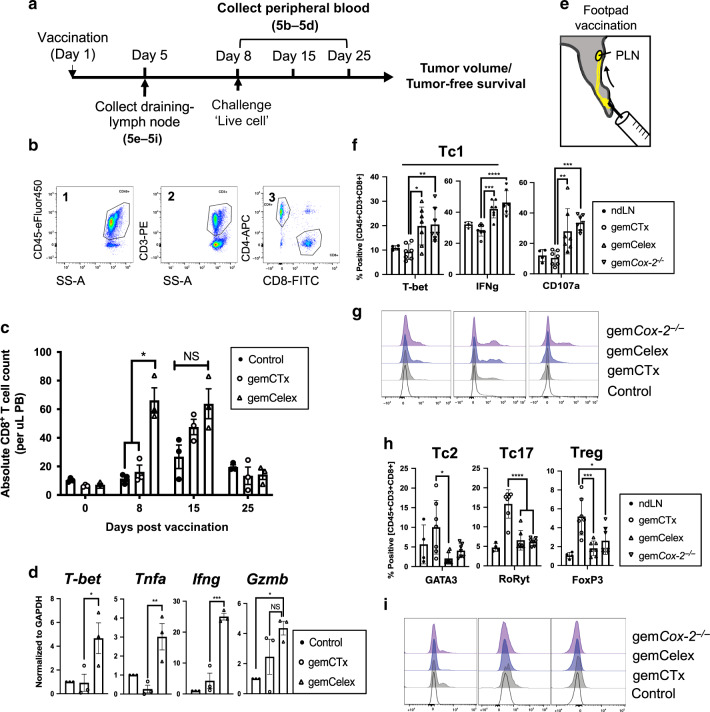
COX-2/PGE_2_ blockade promotes priming of a CD8^+^ Tc1-mediated immune response. **a** A schematic depiction of the experimental procedures implemented to analyze vdLN (i.e., popliteal) and PB-circulating CD8^+^ T cells. **b** Representative gating-strategy implemented to analyze/sort CD8^+^ T cells from vdLN and PB by flow cytometry. **c** Quantification of PB-circulating CD8^+^ T cells post-vaccination (*n* = 3 per treatment group). **d** qPCR analysis of genes associated with functionally activated Tc1-polarized CD8^+^ T cells. **e** An illustration depicting the adapted in vivo vaccination assay to interrogate vdLN CD8^+^ T cells. **f**–**i** Flow cytometry analysis and quantification of vdLN CD8^+^ T cells 5-days post-vaccination (non-dLN *n* = 4; vdLN *n* = 5 per group). PB peripheral blood, PLN popliteal lymph node. Statistics: two-tailed, two-way ANOVA-Tukey’s multiple comparisons test (**c**; *p = 0.387); two-tailed, one-way ANOVA (**d**, *T-bet;* *p* = 0.0483), (**d**, *Tnfa*; *p* = 0.0080), (**d**, *Ifng*; *p* = 0.0002), (**d**, *Gzmb*; *p* = 0.0379), (**f**, T-bet; *p = 0.0136 and **p = 0.0089), (**f**, IFNg; ***p = 0.0008 and ****p < 0.0001), (**f**, CD107a; **p = 0.0071 and ***p = 0.0004), (**h**, GATA3; *p* = 0.0115), (**h**, RoRyt; ****p < 0.0001), and (**h**, Foxp3; *p = 0.0103 and ***p = 0.0008); and where appropriate, data are presented as mean values ±SEM.

Immunogenic cell death is functionally postulated to depend on a cytotoxic CD8^+^ T cell response^27^. Since our results from Fig. 4b–d revealed that COX-2/PGE_2_ blockade favors immunogenic DC maturation, we reasoned that COX-2/PGE_2_ blockade would promote a CD8^+^ cytotoxic T (Tc)-1 cell polarized response. To evaluate this, we isolated and analyzed peripheral blood-circulating CD8^+^ T cells at 0, 8, 15, and 21 days post-vaccination using fluorescence-activated cell sorting (FACS; Fig. 5a, b). The vaccination arms are as indicated in Fig. 5c (*n* = 3 mice per group), following the immunization protocol described in Fig. 2e. The frequency of circulating CD8^+^ T cells in peripheral blood was significantly elevated 8-days post-vaccination in the COX-2/PGE_2_-blockade arm when compared to the other vaccination groups (Fig. 5c). These results implicated CD8^+^ T cells as the effectors for tumor rejection observed in Fig. 3g, h.

To assess whether the increased number of circulating CD8^+^ T cells associated with a Tc1-polarized immune response, we FACS-purified CD8^+^ T cells from 8-days post-vaccination (Fig. 5a, c) and analyzed for Tc1-associated gene expression using RT-qPCR^60^. Remarkably, CD8^+^ T cells isolated from mice injected with the COX-2/PGE_2_-blockade vaccine exhibited elevated mRNA expression of the Tc1 transcription factor *T-bet*, as well as Tc1-associated cytokines *Tnfa* and *Infg*, when compared to the other vaccination groups (Fig. 5d). Granzyme B, a marker of cytotoxic activity, was also elevated in the same CD8^+^ T cells, illustrating COX-2/PGE_2_ blockade effectively induced Tc1-effector genes.

To verify these mRNA expression findings at the protein level, we adapted the vaccination assay and developed another model where the vaccine-draining lymph nodes (vdLN; i.e., popliteal) from murine footpad injections can be collected for flow cytometry analysis. We also developed *Cox-2*^−/−^ G69 cells using CRISPR/Cas9 as a genetic approach to definitively abrogate PGE_2_ biosynthesis (Supplementary Fig. 9). Appropriate vaccines (i.e., control [PBS], gemCTx, gemCelex, and gem*Cox-2*^−/−^) were then injected in the footpad to evaluate their relative mechanistic effects in regulating CD8^+^ T cell function (*n* = 4 for control and *n* = 7 lymph nodes per vaccination group). Five days post-vaccination, the popliteal vdLN were harvested and examined for CD8^+^ T cell polarization using flow cytometry (Fig. 5a, e). Reflective of the transcriptional results in Fig. 5d, there was a statistically significant enrichment of T-bet positive (*p* < 0.05) and IFNγ-producing (*p* < 0.005) CD8^+^ T cells that also displayed enhanced CD107a (an established marker for T cell activity; *p* < 0.005) in the COX-2/PGE_2_-blocked vaccine groups (Fig. 5f, g). Intriguingly, the gemcitabine-only vaccine promoted an enrichment for Tc17/Treg polarization (Fig. 5h, i), where COX-2/PGE_2_-blockade skewed CD8^+^ T-cell polarization toward a Tc1 phenotype (Fig. 5f, g). These gemcitabine-only vaccines did not display CD107a^+^ enrichment (Fig. 5f, g). Moreover, these T-cell polarizing effects were specific to the corresponding vdLNs; we collected the non-draining popliteal lymph nodes (ndLNs) from the opposite limbs and analysis revealed no specific polarization nor T cell activation (Fig. 5f–i). These results underscore the significance of the vaccines in promoting specific T cell response(s). Combined with prior results in Fig. 3g–j, these data implicate ICD-induced antitumoral CD8^+^ T-cell response results from T-cell skewing towards a Tc1 rather than a Tc17/Treg polarization type.

### Immunogenic cell death-induced CD8^+^ T-cell response causes tumor rejection upon challenge

**Fig. 6:**
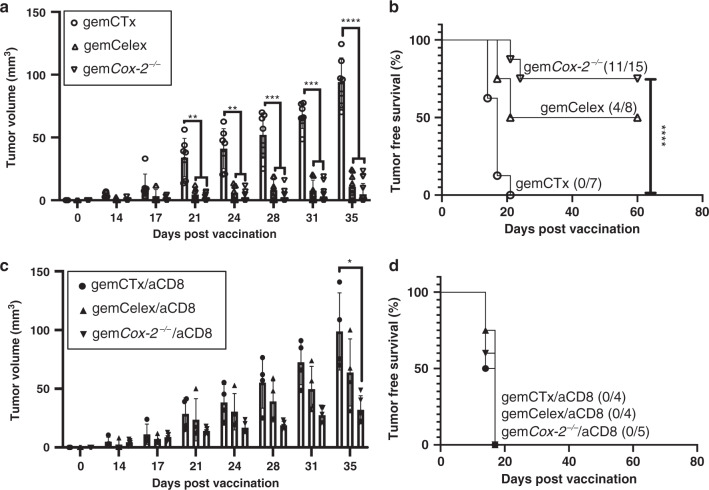
CD8^+^ T-cell-mediated immune response drives tumor rejection upon challenge. Corresponding vaccination assay resulting from two methodologies of iDAMP blockade: pharmacological (i.e., celecoxib) and genetic (CRISPR/Cas9 KO). **a** Tumor volume and **b** tumor-free survival from the same vaccination assay comparing two methods to deplete PGE_2_/iDAMP and their efficacies in affecting drug-induced immunogenic cell death. Corresponding aCD8 mAb treatment results as shown in **c**, **d** Vaccine groups: gem (*n* = 7); gemCelex (*n* = 8); gem*Cox-2*^−/−^ (*n* = 15); gem/aCD8 (*n* = 4); gemCelex/aCD8 (*n* = 4); gem*Cox-2*^−/−^/aCD8 (*n* = 5). Statistics: two-tailed, two-way ANOVA-Tukey’s multiple comparisons test (**a**; **p < 0.0028, ***p < 0.0006, and ****p < 0.0001) and (**c**; *p* = 0.0452); Kaplan–Meier survival analysis using Mantel–Cox test (**b**; *p* < 0.0001); and where appropriate, data are presented as mean values ±SEM.

**Fig. 7:**
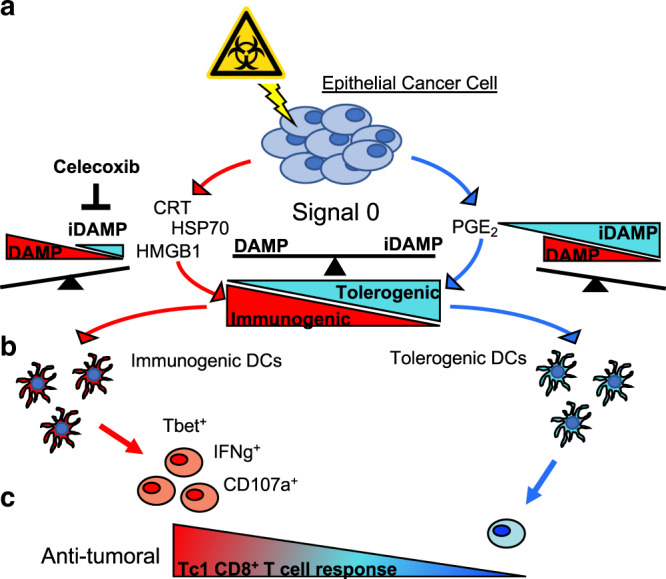
schematic depiction of the proposed immunostimulatory/inhibitory DAMP balance model in modulating drug-induced immunogenic versus tolerogenic cell death fate. **a** The modulation of immunogenic cell death via iDAMP blockade. **b** Signal 0 skews the maturation phenotype of dendritic cells. **c** Resulting polarization and antitumoral CD8^+^ T cell response.

Interferon γ-secreting cytotoxic CD8^+^ Tc1 cells are crucial for host protective antitumoral immunity^61^. Since COX-2/PGE_2_ blockade favored Tc1 polarization (Fig. 5f, g), we next evaluated whether immunogenic cell death-induced tumor rejection was dependent on CD8^+^ T cell activity. To functionally interrogate this, we employed an established protocol of anti-CD8 monoclonal antibody treatment to deplete CD8^+^ cells from vaccine recipient hosts (Supplementary Fig. 10). We again implemented celecoxib (pharmacological) and *Cox-2*^−/−^ (genetic) for COX-2/PGE_2_ blockade. As expected, gemCelex and gem*Cox-2*^−/−^ (iDAMP blockade) vaccine groups resulted favorably with 4/8 (pharmacological) and 11/15 (genetic) mice rejecting tumor engraftment (Fig. 6a, b; statistically significant *p* < 0.05). In the presence of CD8-depletion, however, regardless of the vaccination type, all tumors engrafted and became palpable by 17 days post-challenge (Fig. 6c, d; *n* = 4 or 5 mice per vaccination group, as indicated). Combined with the vdLN data, we demonstrate that immunogenic cell death is dependent on an intricate balance between immunostimulatory and inhibitory DAMPs that converge as signal 0 to promote a type-1-polarized CD103^+^ DC/CD8^+^ T cell immune response, resulting in tumor rejection (Fig. 7).

## Discussion

Gemcitabine—a standard-of-care chemotherapy that is commonly prescribed to treat various solid tumors, including bladder and pancreatic cancers—in facilitating antitumor immunity has long been debated. In the present study, we employed unbiased mass spectrometry proteomics and other molecular approaches to demonstrate that gemcitabine, as monotherapy, can indeed induce the ectopic expression of hallmark DAMPs. These findings were conserved in both human and murine models of bladder cancer, as well as a murine pancreatic ductal adenocarcinoma (PDAC) model. These results contrast a prior study reporting on the inability of gemcitabine to promote cell surface CRT expression^28^.

The current defining molecular feature of ICD includes the ectopic expression or release of hallmark, immunostimulatory DAMPs^3–5,7,31,32^. As a result, anticancer agents that promote successful hallmark DAMP release are anticipated to initiate antitumoral immunity^3–5,7,31,32^. Here, we evaluated the immunogenicity of gemcitabine using independent murine bladder cancer and PDAC as study models. Our results unexpectedly revealed that gemcitabine is unable to propagate proper antitumoral immune response in a gold-standard in vivo vaccination assay. In contrast to conventional wisdom, we found that hallmark DAMPs alone are not the only factor(s) involved in regulating the immunogenicity of drug-induced cell death.

We previously reported on gemcitabine-induced release of PGE_2_, a downstream metabolite derived from arachidonic acid, promotes chemoresistance via inducing the repopulation or compensatory proliferation of residual cancer stem cells^34,62^. Most recently, PGE_2_ was implicated to function as an immunomodulatory inhibitory DAMP (iDAMP) by suppressing macrophage function in the context of mechanical stress^33^. Our results exemplify the immunosuppressive function of this iDAMP as a physiologic response to drug-induced cell death. Furthermore, we identified a regulatory circuit that relies on the intricate balance between immunostimulatory and inhibitory DAMPs to effectively propagate ICD, culminating with antitumoral CD8^+^ T cells. We also functionally demonstrated the pharmacological applicability of tipping the DAMP/iDAMP balance by converting gemcitabine from a non-ICD-inducer into an ICD-inducing drug.

Since the gold-standard in vivo vaccination assay relies on a multimodal, stepwise cascade of immune signaling transduction, to elucidate the mechanistic details, we utilize a bone marrow-derived dendritic cell culture to assess the effects of PGE_2_ on DC activation and maturation. Complimenting the in vivo vaccination assay, BMDCs provided a controlled in vitro system, which allowed us to interrogate the initial immunomodulatory function of dying cell-released PGE_2_. Importantly, our in vitro findings utilizing BMDCs correlated with in vivo data obtained from the vaccination assay, as well as the profiling of peripheral blood-circulating and vaccine-draining lymph node CD8^+^ T cells. Such findings provide new insights into how cell death associated PGE_2_ release precludes successful propagation of an antitumoral Tc1-polarized immunity, in the context of chemotherapy treatment. Clinically, these results also provide a conceptual foundation to explain why other chemotherapies are also non-ICD-inducing in addition to gemcitabine. Such concept is timely and could have important clinical implications, since many ongoing clinical trials are combining chemotherapy with immune checkpoint blockade in attempt to achieve synergistic efficacies. However, these trials have largely resulted with mixed clinical responses depending on the tumor type. In bladder carcinomas, a recent Phase 3 clinical trial combining gemcitabine-based chemotherapy regimen with immune checkpoint blockade failed to meet the expected clinical endpoint^63^, underscoring the clinical pertinence to evaluate COX-2/PGE_2_ blockade as an adjuvant therapy to enhance chemoimmunotherapeutic efficacy.

Indeed, tumor-derived PGE_2_ has been previously reported to promote melanoma tumor growth through immune evasion in a treatment-naive setting^64^, whereas a follow-up study further revealed that the tumor-derived PGE_2_ hindered CD103^+^ DC recruitment into the tumor microenvironment^65^. Furthermore, the genetic ablation of PGE_2_ in melanoma was shown to synergize with PD-1 blockade to enhance antitumoral immunity^64^. These data corroborate the most recent studies indicating the expression of dendritic cell PD-L1 to be the key regulator of T-cell immunity in cancer^66,67^. In the context of chemotherapeutic treatment (i.e., current study), cell death-induced release of PGE_2_ also induces tolerogenic DC maturation, which will likely diminish immune checkpoint response. Since we demonstrated the effectiveness of PGE_2_ blockade in converting a non-ICD-inducing chemotherapy (i.e., gemcitabine) into an ICD-inducer; together with the above studies, we speculate that iDAMP (or PGE_2_) blockade will synergize with immune checkpoint blockade in the context of chemotherapy treatment. To fully evaluate the applicability of iDAMP blockade in a therapeutic context, utilization of preclinical model(s) following standard-of-care treatment regimen is recommended before its advancement into clinical trials. Thus, the functional assessment of PGE_2_ blockade plus chemotherapy will shed light on the immunological response(s) that result from a chronic treatment setting on pre-existing tumors—as opposed to the acute immunological response that ensue from vaccination with dying cancer cells.

Several studies^17,21^ recently documented on the outcome of clinical trials that evaluated the prognostic value of immunostimulatory DAMPs and their association with the therapeutic efficacy of bona fide ICD-inducing chemotherapies^68–71^. These reports focused on common ICD-induced immunostimulatory DAMPs as biomarkers (i.e., HMGB1 and CRT) in various cancer types, including breast and colorectal cancer, as well as AML^68–71^. However, based on our present findings and through the works of others^33,34,64,65^, it seems imperative to consider iDAMP as an additional biomarker; PGE_2_ has been reported to negatively influence the immune landscape in various tumor types^59,60^. Our findings will most definitely impact the current understanding of chemotherapy-induced ICD in the clinical setting.

Here we show that a non-ICD-inducing chemotherapy can be converted into an ICD-inducing drug through PGE_2_ blockade and provide new insight towards the fundamental understanding of immunogenic cell death. Previous high throughput studies only evaluated drug-induced DAMPs as an indicator to determine whether a certain drug is immunogenic or non-ICD-inducing. The evidence of PGE_2_ as an inhibitory DAMP and the likely existence of a whole family of other iDAMPs will provide additional context to prior studies, as well as burgeon new avenues to exploit ICD in the context of immune checkpoint blockade therapies.

## Methods

### Cell culture

The human bladder cancer line, T24, was purchased from ATCC, and was maintained in DMEM (Sigma, D5796) supplemented with 10% fetal bovine serum (Sigma, catalog no. F0926) containing 100 μg/mL of streptomycin and 100 Units/mL of penicillin (GE Healthcare, SV30010). The murine bladder cancer cell line, G69, was generated by our laboratory, and was maintained in DMEM/F12 (Sigma, D6421) supplemented with 10% fetal bovine serum containing d-glucose (Fisher, AAA168280E), 1x sodium pyruvate (Gibco, 160070), 1x glutamax (Gibco, 35050061), 1x insulin-transferrin-selenium (Gibco, 41400045), 100 μg/mL of streptomycin, and 100 Units/mL of penicillin. The Panc02 cells were maintained in DMEM high supplemented with 10% FBS and 1% PS. T cells and bone marrow-derived dendritic cells were maintained in RPMI media (Sigma, R8758) supplemented with 50 μM beta-mercaptoethanol (Santa Cruz Biotechnology, sc-202966), 1x glutamax (Gibco, 35050061), and 10% heat-inactivated fetal bovine serum. Cells were incubated at 37 °C in a humidified atmosphere containing 5% CO_2_. In vitro treatment of cells with chemotherapy were carried out in DMEM high cultured media supplemented with reduced fetal bovine serum concentration of 2%. T24, G69, and Panc02 cells were treated with appropriate IC-50 concentrations of mitoxantrone (Sigma, M6545-10MG), gemcitabine (TCI, 501332958), or cisplatin (Sigma, P4394). Celecoxib (Selleck Chemicals, 50-784-7) treatment of 3 μg/mL was used for all cell lines. At the time of collection, cultured media were first centrifuged at 1200 × *g* for 5 min to ensure the collection of floating cells. Supernatants were then centrifuged at 18,000 × *g* for 15 min at 4 °C to pellet cellular debris. Debris-free supernatants were utilized for downstream ELISA, ATP-Luciferase assay, and western blot analyses. Adherent cells were dissociated using TrypLE express enzyme (Gibco, 12605028), combined with the detached, floating cell pellets, and re-pelleted by centrifugation (1600 × *g* for 5 min at room temperature) for downstream flow cytometric and western blot analyses.

### Mice

Wild-type FVB and C57/Blk6 mice were utilized for experimental purposes. All in vivo experiments used 8- to 12-week-old mice, housed in either Baylor College of Medicine or Cedars-Sinai Medical Center animal facilities. All studies were performed in accordance with procedures approved by the Institutional Animal Care and Use Committee of Baylor College of Medicine and Cedars-Sinai Medical Center.

### Gold-standard in vivo vaccination assay

G69 and Panc02 cells were seeded at 4.7 × 10^4^ cells per mm^2^ and were treated with either cisplatin (negative control), mitoxantrone (positive control), gemcitabine, or gemcitabine plus celecoxib for 24 h in vitro. After 24 h, adherent cells were washed with DPBS (Sigma, catalog no. D8537) thoroughly, dissociated with TrypLE express enzyme and pelleted by centrifugation (1300 × *g* for 5 min at room temperature). Cell pellets were washed with DPBS once more to ensure clearance of residual enzyme and pharmacological agent(s). 5 × 10^5^ cells were suspended in 15 μL of DPBS and were injected either (i) subcutaneously into the left lower flank or *ii)* footpad of mice. A week following vaccination (lower flank vaccination), mice were challenged with 5 × 10^5^ cells that were suspended in 15 μL of DPBS. Mice vaccinated in the footpad were euthanized 5 days post-vaccination and the vaccine-draining lymph nodes (i.e., popliteal) were collected for subsequent analysis. Tumor growth and incidence were recorded twice a week with calipers. Peripheral blood was taken from mice at days 0, 8, 15, and 22 post-vaccination. Tumor volume was calculated using the standard formula (width × length × length/2).

### CD8^+^ cell depletion

Mice were injected subcutaneously with aCD8 mAb (BioXcell, BE0004-1-A005) at a concentration of 200 μg per 30 mg mouse. Mice were injected twice with the aCD8 mAb prior to vaccination: first and second injections were 72 and 48 h prior to live cell challenge, respectively. Efficacy of CD8-depletion was checked prior to challenge using murine tail-vain blood sampling coupled with flow cytometry analysis.

### Processing peripheral blood for flow cytometry

Peripheral blood from mice were taken (40–60 μL) via tail-vein and collected in 1.5 mL BD Microtainer tubes (BD, BD365974). In all, 40 μL of peripheral blood was taken from each sample and subjected to centrifugation at 875 × *g* for 10 min at 4 °C to separate plasma and cells. Red blood cells were lysed using ACK buffer (Lonza, 10548E), and remaining cells were subjected to antibody staining for flow cytometric analyses.

### Processing vaccine-draining lymph nodes for flow cytometry

Vaccine-draining lymph nodes were harvested 5 days post-vaccination of murine foot pad. Non-draining lymph nodes (i.e., opposite foot) were harvested as controls. Harvested lymph nodes were passed through a 70-μ mesh filter. Flow-through immune cells were then centrifuged and incubated in 96-well U-bottom plates coated with aCD3 (BD, 553057) and aCD28 (Thermo, 16-0281-82) with RPMI (50uM BME, 10% heat-inactivated FBS, and 1x activation cocktail) for 6 h. One hour prior to collection, CD107a-PE/Dazzle antibody (diluted to 1:200) was added. T cells were then collected, washed with DPBS, and processed for flow cytometric analysis.

### Generation of bone marrow-derived CD103^+^ dendritic cells

Generation of bone marrow-derived CD103^+^ dendritic cells followed a stepwise protocol previously described by Mayer et al.^41^. In short, bone marrow cells were collected from murine femur and tibia. In all, 15 × 10^6^ bone marrow cells were cultured in RPMI media (Sigma, R8758) supplemented with 50 μM beta-mercaptoethanol (Santa Cruz Biotechnology, sc-202966), 10% heat-inactivated fetal bovine serum, 200 ng/mL of FLT3L (PeproTech, 50399689), and 5 ng/mL of GM-CSF (Miltenyi,130-094-043) for 9 days. On day 6, 5 mL of fully supplemented media was added to cultures. On day 9, 3 × 10^6^ floating cells were re-seeded in a new 10-cm dish with fresh, fully supplemented RPMI culture media and were incubated at 37 °C in a humidified atmosphere containing 5% CO_2_ for an additional 7 days.

### Treating CD103^+^ BMDCs with cultured media

CD103^+^ BMDCs from 16 days of culture were harvested using 5 μM EDTA solution. 1 × 10^4^ cells were suspended in gemCTx pre-treated G69 cultured media (20% of total volume and 80% base DC media as described above) in the absence or presence of iDAMP blockade. Cultured media PGE_2_ sequestration was achieved by treating the cultured media with 100 nM of anti-PGE_2_ mAb (Cayman, 10009814) for at least 30 min on ice. CD103^+^ BMDCs were cultured and collected at 6- and 24-h post activation with appropriate culture medium treatment.

### Flow cytometry/FACS

All immune cell samples were suspended in 50 μL of anti-CD16/32 antibody (BD, BDB553141) solution at a dilution of 1:200 in PBS for 10 min on ice prior to subsequent immunophenotype staining. Immunophenotype staining was performed with antibodies diluted to 1:100 (final concentration). Peripheral blood-circulating cells were stained with the following antibodies: CD45-eFluor 450 (Fisher, 501129701); CD3-PE (Biolegend, 100206); CD4-APC (VWR, NC1556315); and CD8-FITC (Biolegend, 100706). Liquid counting BEADs (335925, BD) were used to quantify the absolute count of peripheral blood-circulating CD8^+^ T cells. Vaccine-vdLN T cells were stained with the following antibodies: CD45-Pacific Blue (Biolegend, 103126), CD3-PE (Biolegend, 100206), CD4-APC (VWR, NC1556315), CD8-FITC (Biolegend, 100706), Tbet-BV711 (Biolegend, 644819), GATA3-PerCP/Cy5.5 (Biolegend, 653812), RoRyt-PerCP/eFluor710 (Thermo 46-6981-82), FoxP3-BV421 (Biolegend, 126419), IFNg-BV785 (Biolegend, 505838), and CD107a-PE/Dazzle (Biolegend, 121624). Intracellular staining was performed using the BD Cytofix/Cytoperm (BD, 554714) following the manufacturer’s protocol. Bone marrow-derived CD103^+^ dendritic cells were stained with either: CD45-eFluor 450 (Fisher, 501129701); B220-APC (Biolegend, 103212); CD11c-PE/Cy7 (Biolegend, 117318); MHCII-BV510 (Fisher, 50402975); XCR1-BV650 (Biolegend, 148220) and CD103-BV786 (BD, BDB564322), or CD45-BV570 (Biolegend, 103135), CD11c-Biotin (Biolegend, 117304), CD11b-APCR700 (BD, BD564985,), CD103-BV711 (Biolegend, 121435), XCR1-BV650 (Biolegend, 148220), H2Kq-AF647 (Biolegend, 115106), MHCII-BV510 (Biolegend, 107636), CD40-FITC (Biolegend, 124608), CD86-PerCP/Cy5.5 (Biolegend, 105028), IL-10-BV421 (Biolegend, 505022), IL-12-PE (Biolegend, 505204), PD-L1-PE/Dazzle (Biolegend, 124324), Streptavidin-PE/Cy5 (Biolegend, 405205), and Live/Dead-NearIR stain (Thermo, L10119). To determine cell-surface DAMP expression, chemotherapy-treated cancer cells were stained with anti-CRT-PE (Cell Signaling, 19780 S) or anti-HSP70-PE (Miltenyi, 130-105-549), using a final concentration of 1:100 and 1:10, respectively, as recommended in the manufacturer’s instructions. Concurrently, these DAMP-stained cells were labeled with a cell viability dye (e.g., DAPI at a 1-μg/mL concentration) for dead cell exclusion. All antibody cocktails were diluted in ice-cold PBS. Cells were incubated with the appropriate antibody cocktails on ice for roughly 20 min and washed thoroughly with ice-cold DPBS. Flow cytometric analysis of immune cells was performed on BD LSRFortessa™ and Cytek™ Northern Lights cell analyzer. FACS was performed on BD Aria™ II. Flow cytometric analysis of cell surface CRT and HSP70 was performed on BD LSRFortessa™ and BD Accuri™ C6. All flow cytometry data were processed using FlowJo software v.10.7.1.

### ATP detection assay

ATP release by cancer cells was performed according to the manufacturer’s protocol (Abcam, catalog no. ab113849).

### ELISA

ELISA analysis of PGE2 (Cayman) release by cancer cells was performed according to the manufacturer’s protocol.

### Western blot

Cell pellets were lysed in RIPA buffer (Emd Millipore, 20–188) with complete protease (Sigma, 11836153001) and phosphatase (Sigma, 04906837001) inhibitor cocktails. Lysates were collected by centrifugation, 18,000 × *g* for 15 min at 4 °C. Protein concentration was measured using Bradford BCA colorimetric assay (Bio-Rad, 5000006). In all, 15 mg of sample lysates were subjected to western blot analysis using 10% Tris-Glycine gel under reducing conditions. Proteins were transferred onto PVDF membranes (Emd Millipore, IPVH00010) and probed with the following primary antibodies: COX-2 [74 kDa] at 1:1000 (Cell Signaling, 12282S); GAPDH [~37 kDa] at 1:2000 (Santa Cruz biotechnology, SC-32233); and HMGB1 [~29 kDa] at 1:1000 (Biolegend, 651402). Secondary antibodies were purchased from the following sources: anti-mouse-HRP at 1:10,000 (Boster, BA1075) and anti-rabbit-HRP at 1:10,000 (Cell Signaling, 7074 S). Western blot bands were visualized using enhanced chemiluminescence system (Thermo, 32106) on autoradiography films (Genesee, 30-507) or the iBright™ CL750 systems. Western blot was quantified using the ImageJ software (ver. 1.5i) or the iBright analysis software (ver. 1.5. 0).

### Analysis of mRNA expression

mRNA from peripheral blood-circulating CD8^+^ T cells and cancer cell cultured media-treated CD103^+^ dendritic cells were extracted using RNA direct lysis buffer containing 10 mM Tris-HCl pH 7.4 (Sigma, T2194), 0.25% Igepal CA-630 (Sigma, I8890), and 150 mM NaCl (Sigma, S5886), which was freshly prepared on the day of experimentation. cDNA was synthesized using Qscript XLT cDNA supermix (Quantabio, 95161-500), in accordance with the manufacturer’s protocol. Synthesized cDNAs were then subjected to pre-amplification using the Ssoadvanced PreAMP master mix (Bio-Rad, 1725160), in accordance with the manufacturer’s protocol. Fluidigm Biomark analysis was performed using the reagents and cassette in accordance to the recommendations made by the manufacturer. Quantitative real-time PCR was performed using iTaq Universal SYBR Green Supermix (Bio-Rad, 172-5121) and Roche LightCycler96 machine. The relative abundance of mRNA was normalized to GAPDH. Primers for qPCR are included in supplementary table 1.

### Mass spectrometry proteomics

Parallel reaction monitoring (PRM) was implemented to detect cell-surface and extracellular DAMPs (i.e., from conditioned medium). Cell-surface proteins were obtained from T24 and G69 cells treated with or without gemCTx for 48 h through mechanical cell lysing, using a dounce homogenizer and subsequent separation via differential centrifugation (i.e., fractionation). The conditioned medium from T24 and G69 cells were collected at 48 h with or without gemCTx treatment, centrifuged to pellet cellular debris (18,000 × *g* for 10 min at 4 °C), and subsequently subjected to mass spectrometric analyses after concentration. We utilized the PRM method using Orbitrap Fusion™ Tribrid™ mass spectrometer. Results obtained were based on unique peptide availability and three or four unique peptides for each target protein was selected for PRM analysis. Pre-selected precursor ions were scanned with a 10-min predicted elution window and isolated by quadrupole followed by collision-induced dissociation MS2 analysis. For relative quantification, the raw spectrum file was crunched to. mgf format by PD1.4 and then imported to Skyline with raw data file. We validated each result by deleting non-identified spectrum and adjusting the AUC range.

### Generation of COX-2 KO cells

G69 cells were transduced with lentiviral packed TLCV2 plasmid obtained from AddGene (AddGene, 87360) and subsequently selected using puromycin. G69.TLCV2 cells were treated with 1 μg/mL doxycycline (Sigma, D9891) in complete culture media 48 h prior to transfection. A COX-2 sgRNA (Sigma, MMPD0000032290) and tracrRNA (Sigma, TRACRRNA05N) was transfected into cells using RNAiMax transfection reagent (Thermo, 13778); instructions were provided by the manufacturer’s protocols. Single cell clones were selected using limiting dilution and confirmed via western blot analysis.

### Statistics

All data were evaluated and graphed using Prism ver. 7 and 8 software (GraphPad). Statistical comparison between control and experimental groups were performed utilizing two-tailed Student’s *t* test, one-way analysis of variance (ANOVA), two-way ANOVA with multiple comparisons test, or Kaplan–Meier survival analysis when appropriate. Quantified data shown were repeated at least three times in independent experiments (unless specified differently). Data shown are represented as mean ± SEM. *P*-value < 0.05 was considered statistically significant.

### Reporting summary

Further information on research design is available in the Nature Research Reporting Summary linked to this article.

## Supplementary information

Supplementary Information

Reporting Summary

## Data Availability

The mass spectrometry data for protein identification have been deposited via the MASSIVE repository (MSV000086386) to the Proteome X change Consortium (http://proteomecentral.proteomexchange.org/) with the data identifier PXD022253, and data using AB Sciex 5600 instrument has been deposited to the Massive repository with identifier MSV000086407 (https://massive.ucsd.edu). The other relevant data supporting the findings in this study are available in the article, Supplementary Information, or from the corresponding author upon reasonable request.
