# Supplementary material for: Tipping the immunostimulatory and inhibitory DAMP balance to harness immunogenic cell death
Source: Nat Commun. 2020 Dec 7;11:6299. doi: 10.1038/s41467-020-19970-9 (PMC7721802; doi:10.1038/s41467-020-19970-9)
Supplement: Supplementary file 1 — Supplementary Information [file 41467_2020_19970_MOESM1_ESM.pdf]

a

CRT (Cell surface)	EPAVYFK
	FYGDEEK
	FYGDEEKDKGLQTSQDAR
	GLDLWQVK
	IDNSQVESGSLEDDWDFLPPKK
	KEEEEAEDKEDDEDKDEDEEDEEDKEEDEEEDVPGQAK
	KEEEEAEDKEDDEDKDEDEEDEEDKEEDEEEDVPGQAKDEL
	KPEDWDEEMDGEWEPPVIQNPEYK
	LKEEEEDKKRKEEEEAEDKEDDEDKDEDEEDEEDKEEDEEEDVPGQAK
	LKEEEEDKKRKEEEEAEDKEDDEDKDEDEEDEEDKEEDEEEDVPGQAKDEL
HSP70 (Cell surface)	ATVEDEKLQGKINDEDKQK
	ETAENYLGHTAK
	KFGDPVVQSDMK
	MNPTNTVFDAK
	MVNHFIAEFK
	QTQTFTTYSDNQPGVLIQVYEGER
	RQAVTNPNNTFYATK
	SLGIETLGGVFTK
HSP90 (Cell surface)	ADLINNLGTIAK
	ALLFVPR
	APFDLFENR
	DQVANSAFVER
	EDQTEYLEER
	EGLELPEDEEEK
	EGLELPEDEEEKK
	EGLELPEDEEEKKK
	EGLELPEDEEEKKKQEEK
	ELHINLIPNK
	ESEDKPEIEDVGSDEEEEKK
	ESEDKPEIEDVGSDEEEEKKDGDK
	FYEQFSK
	GVVDSEDLPLNISR
	HIYYITGETK
	HLEINPDHSIIETLR
	HSQFIGYPITLFVEK
	IRYESLTDPSK
	KHLEINPDHSIIETLR
	LGLGIDEDDPTADDTSAAVTEEMPPLEGDDDTSR
	NPDDITNEEYGEFYK
	RAPFDLFENR
	SLIINTFYSNK
	SLTNDWEDHLAVK
	TLTIVDTGIGMTK
	YESLTDPSK
	YESLTDPSKLD
	YESLTDPSKLDSGK
	YIDQEELNK
HMGP (Released)	GRPKKLEKEEEEGISQESSEEEQ
	KLEKEEEEGISQESSEEEQ

b

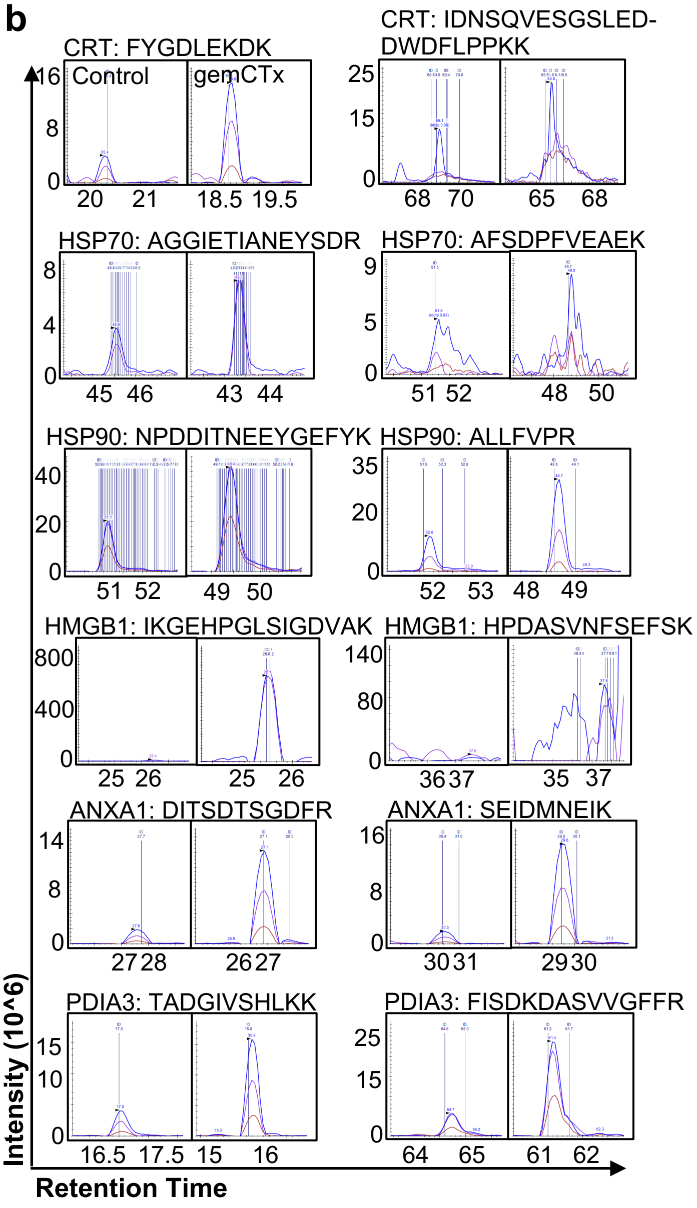

Supplementary Figure 1: MS profiling of hallmark DAMPs. (**a**) List of unique peptide sequences of classical DAMPs enriched in T24 cells (gemCTx- versus non-treated control). (**b**) Representative peptide chromatograms depicting differential enrichment of additional peptide sequences pertaining to hallmark DAMPs released by G69 cells (gemCTx- versus non-treated control; n = two independent studies). Abbreviations: annexin A1 (ANXA1); calreticulin (CRT); gemcitabine chemotherapy-treated (gemCTx); high-mobility group protein box 1 (HMGB1); heat-shock protein (HSP); and protein disulfide isomerase A3 (PDIA3). Extracted chromatograms for a representative peptide of each gene using Skyline software; precursor (blue color), precursor [M+1] (purple color), precursor [M+2] (red color) represent the three isotopic peaks for the indicated peptide.

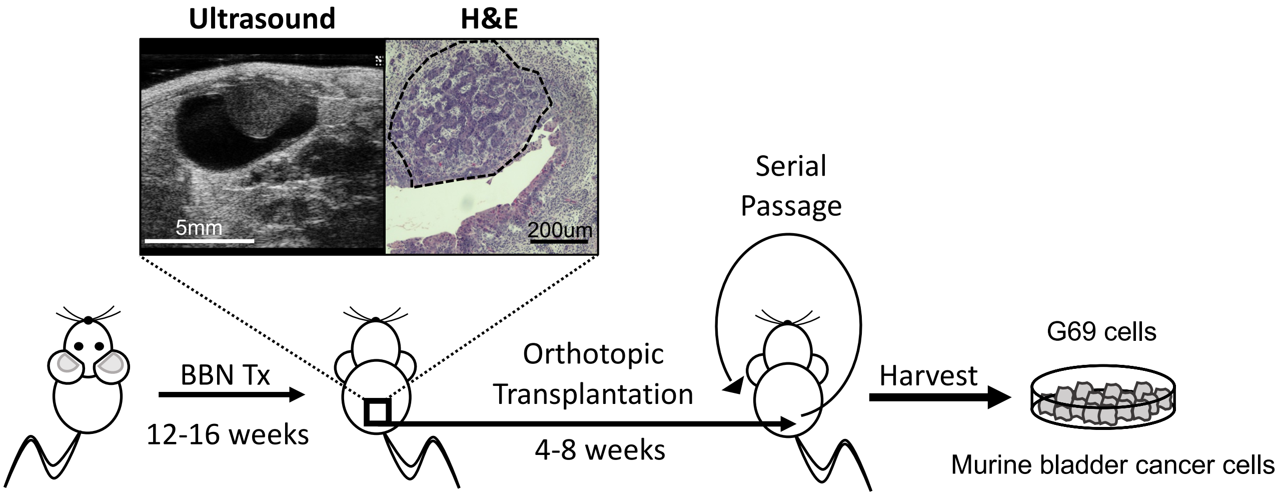

Supplementary Figure 2: A schematic depicting the methodology implemented to generate murine bladder cancer cell lines using a chemically-induced carcinogenesis model. Abbreviations: N-butyl-N-(4-hydroxybutyl)-nitrosamine (BBN); treatment (Tx); Hematoxylin and Eosin (H&E) stain. Representative ultrasound image (bar scale: 5 millimeter) and a 10x magnification H&E stain of BBN-treated murine bladder sections (bar scale: 200 micrometer).

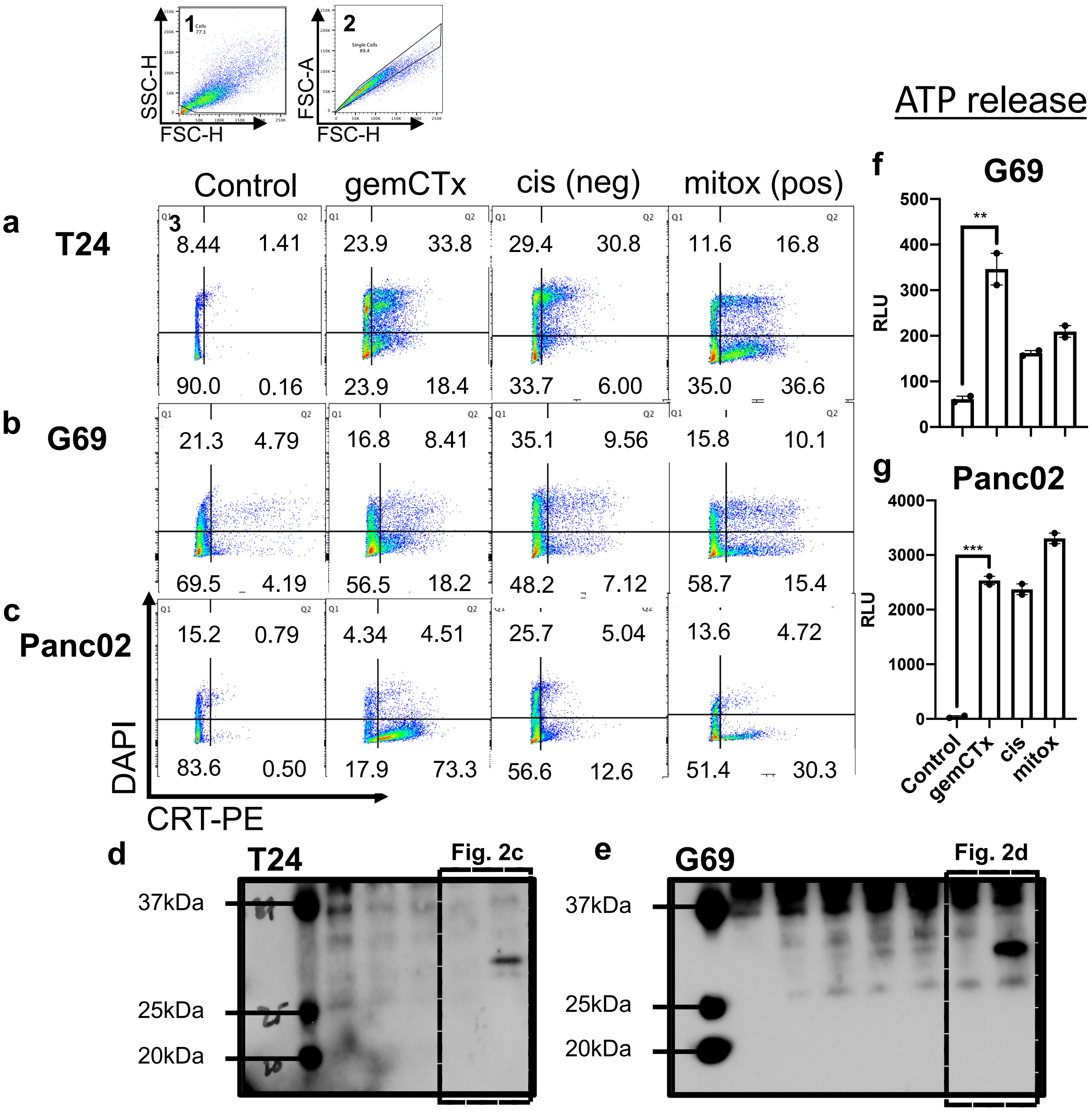

Supplementary Figure 3: Gemcitabine potentiates cell surface CRT, as well as HMGB1 and ATP release. (**a**) Human bladder cancer (T24), (**b**) murine bladder cancer (G69), and (**c**) murine PDAC (Panc02) models were treated with gemcitabine *in vitro* and analyzed for cell surface CRT expression, in conjunction with cell permeability (i.e., DAPI) on single cells using flow cytometry. Representative plot shown (n = 3 independent experiments). Culture media from gemCTx-treated murine (**d**) T24 and (**e**) G69 cells were analyzed for HMGB1 release (uncropped blot shown; n = at least 3 independent experiments). Culture media from gemCTx-treated murine (**f**) G69 and (**g**) Panc02 cells were analyzed for ATP release (representative plot shown with two technical replicates of n = 3 independent experiments). Abbreviations: cisplatin (cis); gemcitabine chemotherapy (gemCTx); mitoxantrone (mitox); and relative light units (RLU). Statistics: Two-tailed, One-way ANOVA-Tukey's multiple comparisons test (d; $p < 0.0016$) and (e; $p < 0.0001$); data are presented as mean values +/- SEM.

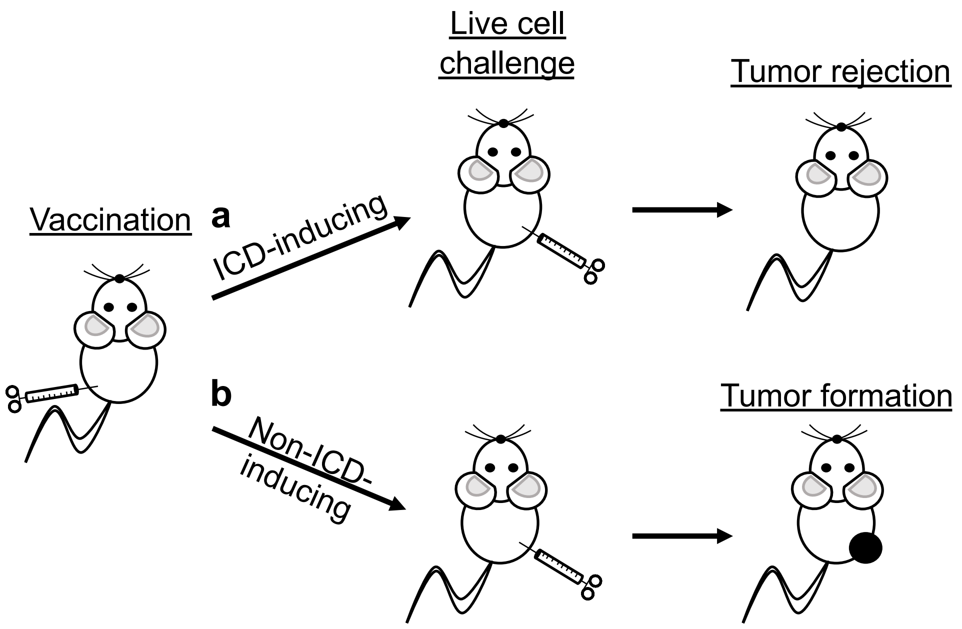

Supplementary Figure 4: A schematic depicting the underlying principle of the *in vivo* gold-standard vaccination assay. (**a**) Mice that are vaccinated with cancer cells pretreated with an ICD-inducing drug (e.g., mitoxantrone) properly immunizes mice, culminating with tumor rejection upon challenge. (**b**) However, mice that are vaccinated with non-ICD-inducing (e.g., cisplatin) vaccines fail to immunize mice; thus, resulting in tumor engraftment.

COX-2 (~74kDa; top)
GAPDH (~37kDa; bottom)

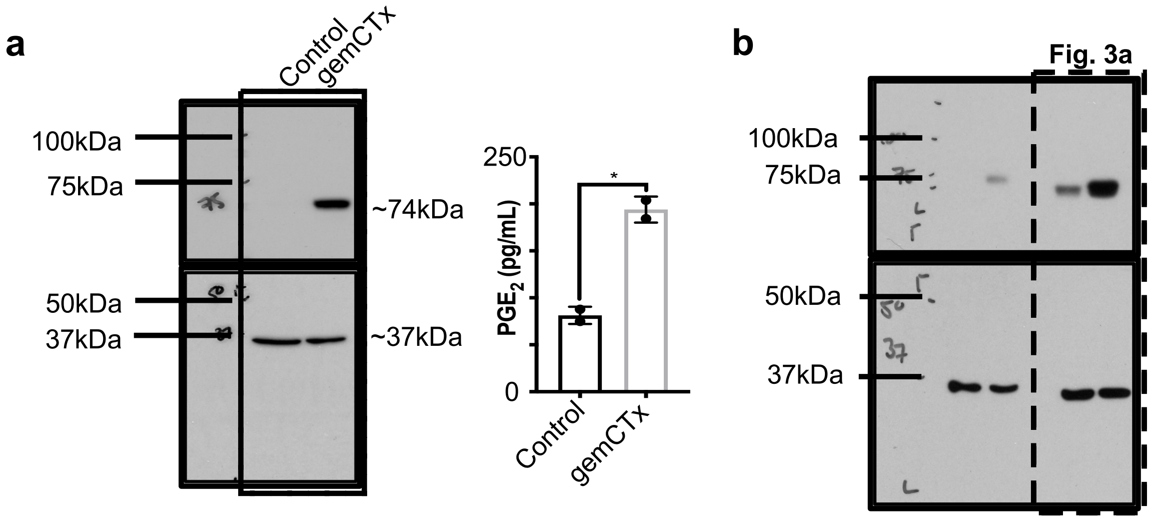

Supplementary Figure 5: Increased COX-2/PGE2 axis in T24 cells treated with gemcitabine. (**a**) COX-2 western blot (uncropped) and PGE_2 ELISA of T24 cells treated with gemcitabine for 48 hrs *in vitro* (representative plot shown with two technical replicates of n = 3 independent experiments). (**b**) COX-2 western blot (uncropped) ELISA of G69 cells treated with gemcitabine for 48 hrs *in vitro.* Statistics: two-tailed, unpaired T test (p = 0.0107); and data is presented as mean values +/- SEM.

COX-2 (~74kDa; top)
GAPDH (~37kDa; bottom)
HMGB1 (~29kDa)

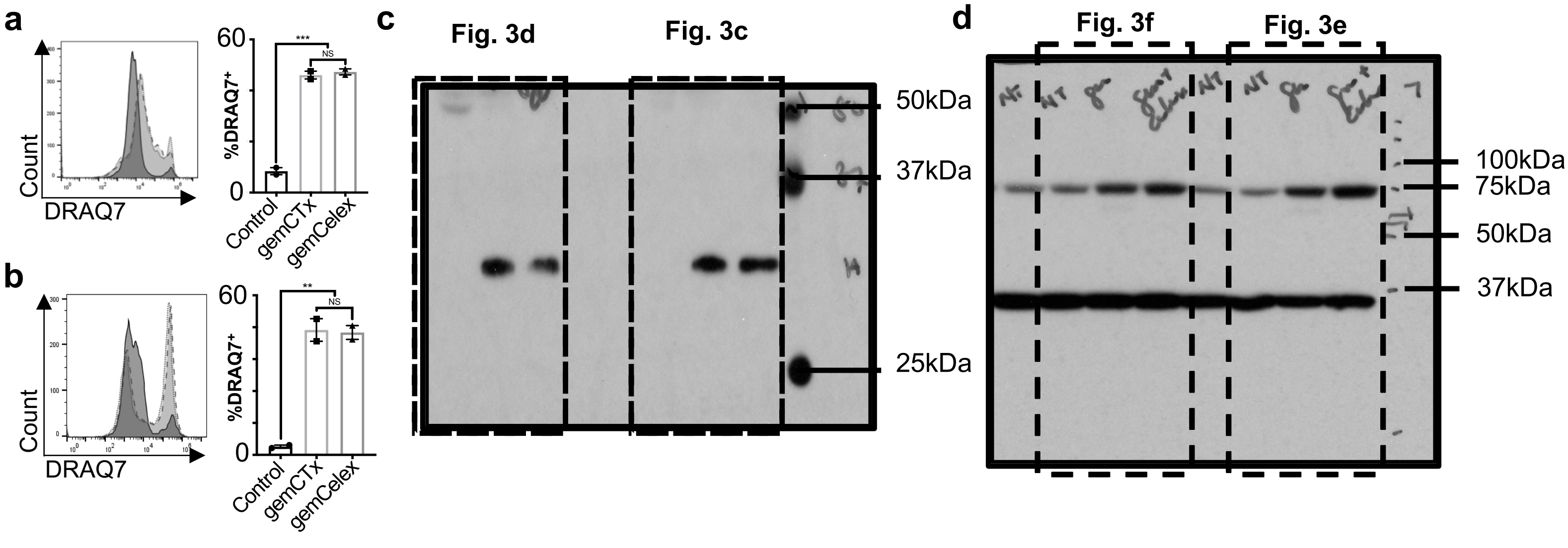

Supplementary Figure 6: iDAMP blockade does not affect cell death nor HMGB1 release. Cell death of (**a**) G69 and (**b**) Panc02 cells measured using DRAQ7 stain on single cells (gating shown in Supplementary Fig. 3) using flow cytometry analysis (representative plot shown with two technical replicates of n = 3 independent experiments). (**c**) HMGB1 and (**d**) COX-2 western blot (uncropped) from cultured media and cell pellets of gemcitabine treated cancer cells (representative plot shown; n = 3 independent experiments). Statistics: two-tailed, one-way ANOVA-Tukey's multiple comparisons test (a; $p = 0.0007$) and (b; $p = 0.0037$); and data is presented as mean values +/- SEM.

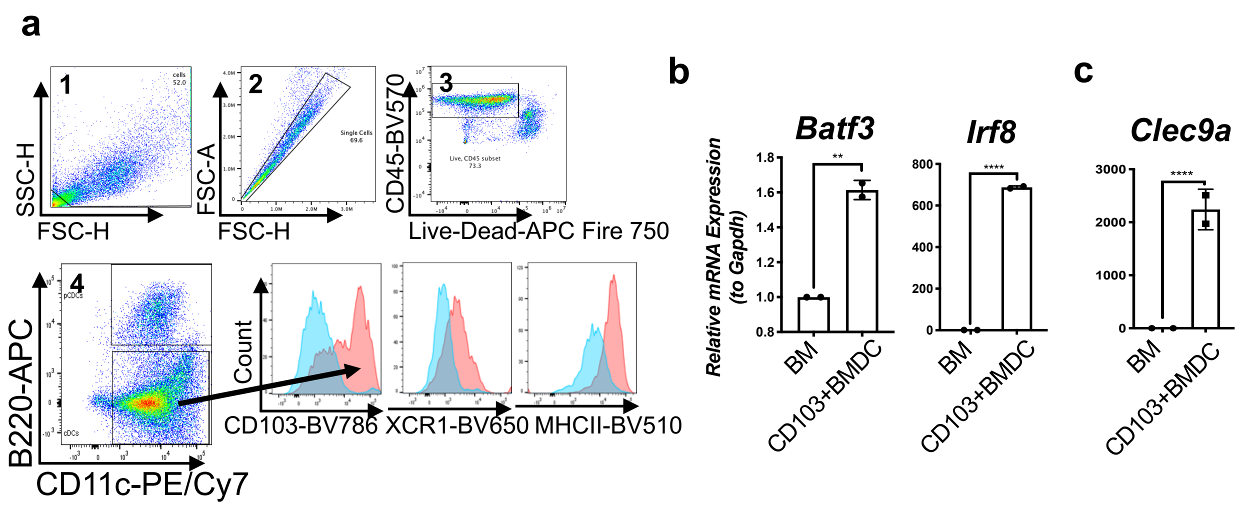

Supplementary Figure 7: Generation of CD103⁺ BMDCs. (**a**) Immunophenotype of CD11c⁺B220⁻ BMDCs after 16 days of culture as previously described by Mayer *et al.*[1] RT-qPCR analysis of conventional DC 1 (cDC1) (**b**) transcription factors *Batf3* ($p = 0.004$) and *Irf8* ($p < 0.0001$) and (**c**) hallmark cDC1 receptor *Clec9a* ($p < 0.0001$). Relative mRNA expression to *Gapdh* and normalized to bulk bone marrow (BM; representative plot shown of two technical replicates of $n = 3$ independent experiments). Statistics: Two-tailed, T test; and where appropriate, data are presented as mean values +/- SEM.

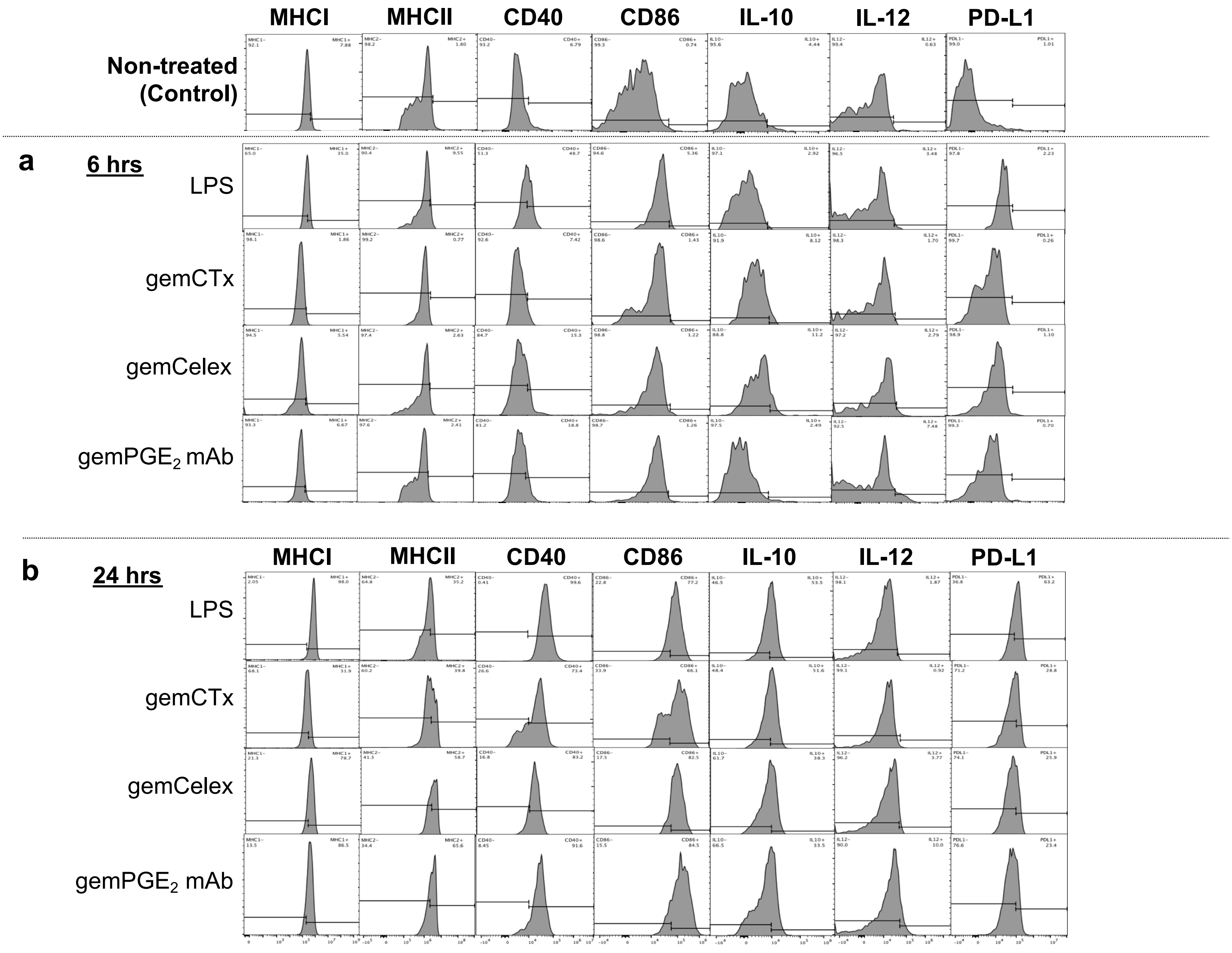

Supplementary Figure 8: CD103$^+$ BMDC immunophenotype post-6 and -24hrs CM treatment. Representative (n = 3 independent experiments) flow cytometry analysis of CD103$^+$ BMDCs (gated shown in Supplementary Fig. 7) activated with gemcitabine-treated G69 cultured media for (**a**) 6 hours or (**b**) 24 hours. Immunophenotype panel included the following (in addition to gating strategy shown in supplementary Fig. 7): MHCI and MHCII (signal I); CD40 and CD86 (signal II); IL-10 and IL-12 (signal III); and last, PD-L1 (inhibitory signal). Representative plot shown (n = 3 independent experiments).

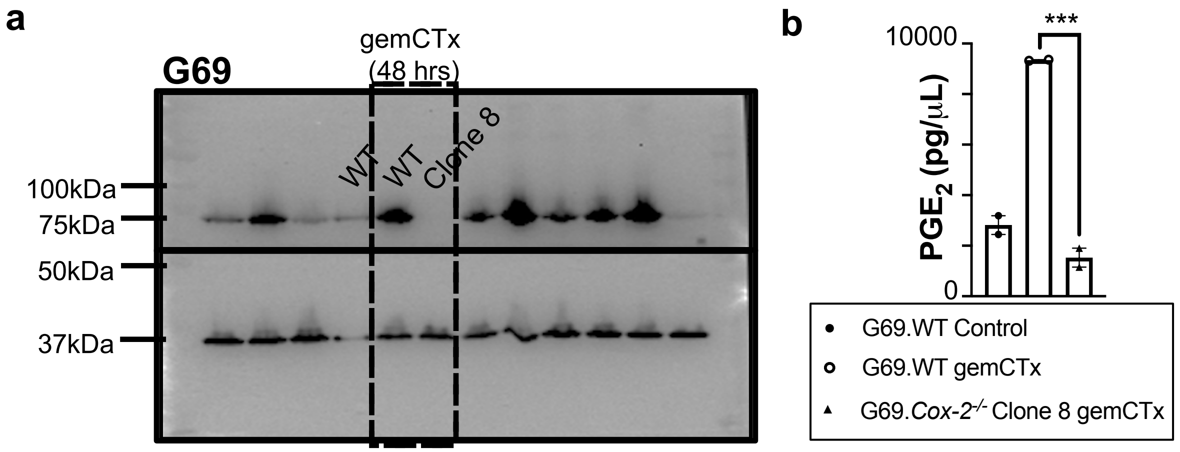

Supplementary Figure 9: (**a**) Generation of *Cox-2*$^{-/-}$ G69 cells using CRISPR/Cas9 as a complementary iDAMP blockade approach (uncropped blot shown). (**b**) PGE_2 ELISA to confirm attenuation of PGE_2 biosynthesis in *Cox-2* KO cells (representative plot shown; n = 3 independent experiments). Statistics: two-tailed, one-way ANOVA-Tukey's multiple comparisons test (***p = 0.006).

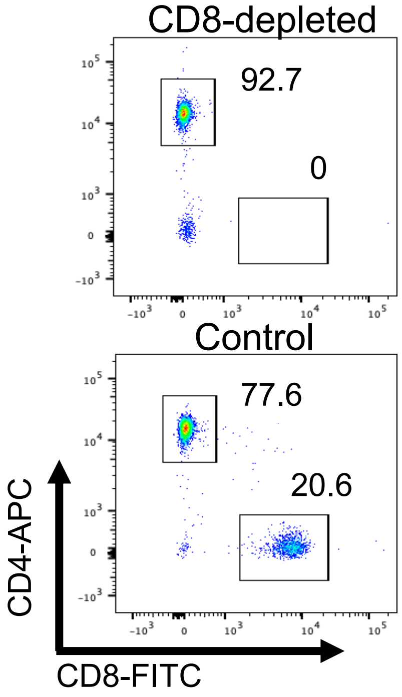

Supplementary Figure 10: Flow cytometry analysis of CD8$^+$ T cells (gating strategy shown in Fig. 5). Utilization of aCD8 mAb to deplete CD8$^+$ T cells in mice prior to vaccination (representative flow cytometry plot shown; n = 3 independent biological replicates).

Mouse	Forward 5' → 3'	Reverse 5' → 3'
Arg1	CTCCAAGCCAAAGTCCTTAGAG	AGGAGCTGTCATTAGGGACATC
Batf3	CAGAGCCCCAAGGACGATG	GCACAAAGTTCATAGGACACAGC
Cd40	TGTCATCTGTGAAAAGGTGGTC	ACTGGAGCAGCGGTGTTATG
Cd80	GCAGGATACACCACTCCTCAA	AAAGACGAATCAGCAGCACAA
Cd83	CGCAGCTCTCCTATGCAGTG	GTGTTTTGGATCGTCAGGGAATA
Cd86	TGTTTCCGTGGAGACGCAAG	TTGAGCCTTTGTAAATGGGCA
Ctla4	AGAACCATGCCCGGATTCTG	CATCTTGCTCAAAGAAACAGCAG
Gapdh	CTCCCACTCTTCCACCTTCG	CCACCACCCTGTTGCTGTAG
Gzmb	CCACTCTCGACCCTACATGG	GGCCCCCAAAGTGACATTTATT
H2-k	GAGACACAGGTCGCCAAGAAC	CGCTGGTAAGTGTGAGAGCC
IcosL	TAAAGTGTCCCTGTTTTGTGTCC	ATTGCACCGACTTCAGTCTCT
Il-2	GTGCTCCTTGTCAACAGCG	GGGGAGTTTCAGGTTCCTGTA
Il-6	ACGGCTTCAGGAAGGTGATG	AGCATCCTGGTGTGTTAGGC
Il-10	GCTCTTACTGACTGGCATGAG	CGCAGCTCTAGGAGCATGTG
Il-12b	TGGTTTGCCATCGTTTTGCTG	ACAGGTGAGGTTCACTGTTTCT
Ido1	GCTTTGCTCTACCACATCCAC	CAGGCGCTGTAACCTGTGT
Ifng	ACAGCAAGGCGAAAAAGGATG	TGGTGGACCACTCGGATGA
Irf8	CGGGGCTGATCTGGGAAAAT	CACAGCGTAACCTCGTCTTC
Pd-l1	TGCTGCATAATCAGCTACGG	GCTGGTCACATTGAGAAGCA
T-bet	AGCAAGGACGGCGAATGTT	GGGTGGACATATAAGCGGTTC
Tim3	TCAGGTCTTACCCTCAACTGTG	GGCATTCTTACCAACCTCAAACA
Tnfa	TAGCCCACGTCGTAGCAAAC	TGTCTTTGAGATCCATGCCGT

Supplementary Table 1. Primers for quantitative Real-time PCR

Supplementary Reference:

1. Mayer, C. T. et al. Selective and efficient generation of functional Batf3-dependent CD103+ dendritic cells from mouse bone marrow. Blood (2014) doi:10.1182/blood-2013-12-545772.
