# Supplementary material for: Tipping the immunostimulatory and inhibitory DAMP balance to harness immunogenic cell death
Source: Nat Commun. 2020 Dec 7;11:6299. doi: 10.1038/s41467-020-19970-9 (PMC7721802; doi:10.1038/s41467-020-19970-9)
Supplement: Supplementary file 2 — Reporting Summary [file 41467_2020_19970_MOESM2_ESM.pdf]

nature research

Corresponding author(s): Keith Syson Chan

Last updated by author(s): Nov 2, 2020

Reporting Summary

Nature Research wishes to improve the reproducibility of the work that we publish. This form provides structure for consistency and transparency in reporting. For further information on Nature Research policies, see our Editorial Policies and the Editorial Policy Checklist.

Statistics

For all statistical analyses, confirm that the following items are present in the figure legend, table legend, main text, or Methods section.

n/a	Confirmed	
☐	☒	The exact sample size (n) for each experimental group/condition, given as a discrete number and unit of measurement
☐	☒	A statement on whether measurements were taken from distinct samples or whether the same sample was measured repeatedly
☐	☒	The statistical test(s) used AND whether they are one- or two-sided<br>*Only common tests should be described solely by name; describe more complex techniques in the Methods section.*
☒	☐	A description of all covariates tested
☒	☐	A description of any assumptions or corrections, such as tests of normality and adjustment for multiple comparisons
☐	☒	A full description of the statistical parameters including central tendency (e.g. means) or other basic estimates (e.g. regression coefficient) AND variation (e.g. standard deviation) or associated estimates of uncertainty (e.g. confidence intervals)
☐	☒	For null hypothesis testing, the test statistic (e.g. F, t, r) with confidence intervals, effect sizes, degrees of freedom and P value noted<br>*Give P values as exact values whenever suitable.*
☒	☐	For Bayesian analysis, information on the choice of priors and Markov chain Monte Carlo settings
☒	☐	For hierarchical and complex designs, identification of the appropriate level for tests and full reporting of outcomes
☒	☐	Estimates of effect sizes (e.g. Cohen's d, Pearson's r), indicating how they were calculated

Our web collection on statistics for biologists contains articles on many of the points above.

Software and code

Policy information about availability of computer code

Data collection	BD LSRFortessa™, Cytek™ Northern Lights, BD Aria™ II, BD Accuri™ C6, iBright CL750
Data analysis	ImageJ ver. 1.5i; Prism ver. 7 & 8; FlowJo ver. 10.7.1.; iBright Analysis Software ver. 1.5.0

For manuscripts utilizing custom algorithms or software that are central to the research but not yet described in published literature, software must be made available to editors and reviewers. We strongly encourage code deposition in a community repository (e.g. GitHub). See the Nature Research guidelines for submitting code & software for further information.

Data

Policy information about availability of data

All manuscripts must include a data availability statement. This statement should provide the following information, where applicable:

- Accession codes, unique identifiers, or web links for publicly available datasets
- A list of figures that have associated raw data
- A description of any restrictions on data availability

The mass spectrometry data for protein identification have been deposited via the MASSIVE repository (MSV000086386) to the Proteome X change Consortium (http://proteomecentral.proteomexchange.org/cgi/GetDataset?ID=PXD022253; dataset identifier PXD022253). Other relevant data supporting the findings are available in the Article, supplementary Information or from the corresponding author upon reasonable request.

Field-specific reporting

Please select the one below that is the best fit for your research. If you are not sure, read the appropriate sections before making your selection.

[x] Life sciences [ ] Behavioural & social sciences [ ] Ecological, evolutionary & environmental sciences

For a reference copy of the document with all sections, see nature.com/documents/nr-reporting-summary-flat.pdf

Life sciences study design

All studies must disclose on these points even when the disclosure is negative.

Sample size	Sample size was calculated using a pilot vaccination assay: with a two-tailed alpha of 0.95 and power of 90%, each vaccination group required at least 5 mice (using G69 as the model)
Data exclusions	no data were excluded from the analysis
Replication	The data presented (if not biologically replicated), were replicated three times (at least) independently. The data provided in the manuscript are representative and reflect the reproducibility of the experiments. All replication attempts were successful.
Randomization	Mice were initially randomized using an online software prior to vaccination
Blinding	Measurements of tumors were conducted by a blinded researcher that had no prior knowledge of vaccine group stratifications.

Reporting for specific materials, systems and methods

We require information from authors about some types of materials, experimental systems and methods used in many studies. Here, indicate whether each material, system or method listed is relevant to your study. If you are not sure if a list item applies to your research, read the appropriate section before selecting a response.

Materials & experimental systems

n/a	Involved in the study	
[ ]	[x]	Antibodies
[ ]	[x]	Eukaryotic cell lines
[x]	[ ]	Palaeontology and archaeology
[ ]	[x]	Animals and other organisms
[x]	[ ]	Human research participants
[x]	[ ]	Clinical data
[x]	[ ]	Dual use research of concern

Methods

n/a	Involved in the study	
[x]	[ ]	ChIP-seq
[ ]	[x]	Flow cytometry
[x]	[ ]	MRI-based neuroimaging

Antibodies

Antibodies used	Flow Cytometry/FACS: aCD3 (BD, 553057), aCD28 (Thermo, 16-0281-82),CD45-eFluor 450 (Fisher, 501129701); CD3-PE (Biolegend, 100206); CD4-APC (VWR, NC1556315), CD8-FITC (Biolegend, 100706),CD45-Pacific Blue (Biolegend, 103126), CD3-PE (Biolegend, 100206), CD4-APC (VWR, NC1556315), CD8-FITC (Biolegend, 100706), Tbet-BV711 (Biolegend, 644819), GATA3-PerCP/Cy5.5 (Biolegend, 653812), RoRyt-PerCP/eFluor710 (Thermo 46-6981-82), FoxP3-BV421 (Biolegend, 126419), IFNg-BV785 (Biolegend, 505838), CD107a-PE/Dazzle (Biolegend, 121624),B220-APC (Biolegend, 103212); CD11c-PE/Cy7 (Biolegend, 117318); MHCII-BV510 (Fisher, 50402975); XCR1-BV650 (Biolegend, 148220), CD103-BV786 (BD, BDB564322), CD45-BV570 (Biolegend, 103135), CD11c-Biotin (Biolegend, 117304), CD11b-APCR700 (BD, BD564985,), CD103-BV711 (Biolegend, 121435), XCR1-BV650 (Biolegend, 148220), H2Kq-AF647 (Biolegend, 115106), MHCII-BV510 (Biolegend, 107636), CD40-FITC (Biolegend, 124608), CD86-PerCP/Cy5.5 (Biolegend, 105028), IL-10-BV421 (Biolegend, 505022), IL-12-PE (Biolegend, 505204), PD-L1-PE/Dazzle (Biolegend, 124324), Streptavidin-PE/Cy5 (Biolegend, 405205), anti-CRT-PE (Cell Signaling, 19780S), anti-HSP70-PE (Miltenyi, 130-105-549).<br>Western Blot: COX-2 [~74kDa] at 1:1,000 (Cell Signaling, 12282S); GAPDH [~37kDa] at 1:2,000 (Santa Cruz biotechnology, SC-32233); and HMGB1 [~29kDa] at 1:1,000 (Biolegend, 651402). Secondary antibodies were purchased from the following sources: anti-mouse-HRP at 1:10,000 (Boster, BA1075) and anti-rabbit-HRP at 1:10,000 (Cell Signaling, 7074S).
Validation	All the antibodies are from commercial sources and have been validated by the vendors and their validation data are available on the manufacturer's website

Eukaryotic cell lines

Policy information about cell lines

Cell line source(s)	T24 (ATCC), Panc02 (gifted by Dr. Cathy Yao, purchased from Covance), G69 (generated in-house)

Authentication	T24 and Panc02 lines were verified by STR
Mycoplasma contamination	All cell lines tested negative for mycoplasma
Commonly misidentified lines (See ICLAC register)	No cell lines used are commonly misidentified in this study.

Animals and other organisms

Policy information about studies involving animals; ARRIVE guidelines recommended for reporting animal research

Laboratory animals	FVB and C57/Blk6 strain mice were used: both male and female ranging between 8-12 weeks of age; all mice were maintained in ambient room temperature (22 +/- 2 C) with humidity of 40~60% and light/dark cycle of 12h/12h.
Wild animals	no wild animals were used
Field-collected samples	there are no field-collected samples
Ethics oversight	All experiments performed were in accordance with procedures approved by the Institutional Animal Care and Use Committee of Baylor College of Medicine and Cedars-Sinai Medical Center.

Note that full information on the approval of the study protocol must also be provided in the manuscript.

Flow Cytometry

Plots

Confirm that:

- [x] The axis labels state the marker and fluorochrome used (e.g. CD4-FITC).
- [x] The axis scales are clearly visible. Include numbers along axes only for bottom left plot of group (a 'group' is an analysis of identical markers).
- [x] All plots are contour plots with outliers or pseudocolor plots.
- [x] A numerical value for number of cells or percentage (with statistics) is provided.

Methodology

Sample preparation	All immune cell samples (e.g., peripheral blood and vdLN immune cells processed using ACK lysis buffer prior to immunophenotyping) were suspended in 50uL of anti-CD16/32 antibody (BD, BDB553141) solution at a dilution of 1:200 for 10 min on ice prior to subsequent to antibody staining. Immunophenotype staining was performed with antibodies diluted to 1:100 (final concentration) in PBS, on ice for approximately 20 min. Live/Dead-NearIR stain (Thermo, L10119) was used to exclude dead cells during analysis. Cancer cell lines from in vitro experiments were stained with either anti-CRT-PE (Cell Signaling, 19780S) or anti-HSP70-PE (Miltenyi, 130-105-549), using a final concentration of 1:100 and 1:10, respectively, as recommended in the manufacturer's instructions. These cells were stained with DAPI at a final concentration of 1ug/mL in FACS buffer.
Instrument	BD LSRFortessa™, Cytek™ Northern Lights, BD Aria™ II, and BD Accuri™ C6.
Software	Flow cytometry data were analyzed using FlowJo v.10
Cell population abundance	Initial assessment of sort-purity was confirmed via re-run of sorted samples on same gating parameters. Sorted samples were identified to have >95% purity.
Gating strategy	Gating strategy (basic): all samples were gated on FSC/SSC and FSC-H/FSC-A.<br>Gating strategy (BMDCs): samples were gated on CD45+/exclusion dye(neg), B220(neg)/CD11c(pos), and subsequently for CD103, XCR1, MHCI, MHCII, CD40, CD86, IL-10, IL-12, and PD-L1.<br>Gating strategy (CD8+ T cells): samples were gated on CD45+/exclusion dye(neg), CD3(pos), CD4(neg)/CD8(pos), and subsequently for Tbet, IFNg, CD107a, Gata3, RORyt, and FoxP3.

- [x] Tick this box to confirm that a figure exemplifying the gating strategy is provided in the Supplementary Information.
